# Supplementary material for: Protocol for a feasibility randomised controlled trial of the ‘Outdoor’ mobility intervention for older adults after hip fracture
Source: PLoS One. 2024 Aug 12;19(8):e0306871. doi: 10.1371/journal.pone.0306871 (PMC12139654; doi:10.1371/journal.pone.0306871)
Supplement: S2 File — (PDF) [file pone.0306871.s002.pdf]

1 Protocol details

1.1 Protocol Title:

Outdoor Mobility After Hip Fracture: A Feasibility Randomised Controlled Trial

1.2 Protocol Version Control

Version number: 2.0
Final: Final
Date: 10-11-2023

1.3 Names (titles), roles and contact details:

Sponsor
Name of Sponsoring Organisation: King's College London

Co-Sponsor
Name of co-sponsoring Organisation/s: NHS Norfolk and Waveney Integrated Care Board

Chief Investigator
Name: Dr Emma Godfrey

Name and address of Co-Investigator(s)
Name: Professor Katie Sheehan (Co-lead)

Name: Professor Catherine Sackley

Name: Professor Sallie Lamb

Name: Professor Siobhan Creanor

Name: Professor Clare Hulme

Name: Professor Finbarr Martin

Name: Professor Toby Smith

Senior Statistician
Name: Professor Siobhan Creanor

2 CI Signature

The Chief Investigator and the RGO (sponsor office) have discussed this protocol. The investigators agree to perform the investigations and to abide by this protocol.

The investigator agrees to conduct the trial in compliance with the approved protocol, GCP, the UK General Data Protection Regulation and Data Protection Act (2018), the Trust Information Governance Policy (or other local equivalent), the UK Policy Framework for Health and Social Care Research, the Sponsor's SOPs, and other regulatory requirements as required.

Chief investigator

Dr Emma Godfrey

	Signature	Date

Contents

1 Protocol details 1
1.1 Protocol Title: 1
1.2 Protocol Version Control 1
1.3 Names (titles), roles and contact details: 1
2 CI Signature 2
Contents 3
3 List of Abbreviations and Definitions 7
4 Summary/Synopsis 8
5 Introduction 10
5.1 The problem 10
5.2 Important of research to patients and health care services 10
5.3 Previous evidence 11
5.3.1 Guidelines and research priorities 11
5.3.2 Systematic reviews of outdoor mobility interventions 11
5.3.3 Summary 13
6 Trial objectives and purpose 13
6.1 Research hypothesis 13
6.2 Primary objectives 14
6.3 Secondary objectives 14
7 Trial design & Flowchart 14
7.1 Trial Design 14
7.2 Study Setting 15
8 Participant selection 15
8.1 Participant inclusion criteria 15
8.2 Participant exclusion criteria 15

8.3 Professional eligibility criteria 16
9 Trial procedures 16
9.1 Participant recruitment 16
9.1.1 Participants 16
9.1.2 Professionals 17
9.2 Screening Procedures 17
9.3 Randomisation Procedures 17
9.4 Blinding 18
9.5 Intervention Procedures 18
9.5.1 Support from existing evidence 18
9.5.2 Theoretical frameworks 18
9.5.3 Development 19
9.5.4 Programme theories 19
9.5.5 Intervention Description 19
9.5.6 Usual care 21
9.5.7 Outdoor intervention -example of schedule 22
9.6 Schedule of Assessments for each visit 24
9.7 Follow up Procedures. 26
9.8 Modifications or discontinuation of intervention 26
9.9 Withdrawal / dropout of participants 26
9.10 End of Trial Definition 27
10 Regulatory Approvals 27
10.1 Amendments 27
10.2 End of Study Reporting 27
10.2.1 End of Study Declaration 27
10.2.2 End of Study Reporting 28

11 Safety & Adverse Events Reporting 28
11.1 Assessment of Safety 28
11.2 Urgent Safety Measures 30
12 Data 30
12.1 Data to be collected 30
12.2 Data handling and record keeping 31
12.2.1 Electronic database 31
12.2.2 Paper-based data 32
12.2.3 Qualitative data 32
12.3 Access to the dataset 33
13 Statistical considerations 33
13.1 Sample size 33
13.2 Statistical analysis 34
13.3 Interim analysis and data monitoring 35
13.3.1 Discontinuation rules and breaking of randomisation code 35
13.3.2 Monitoring, quality control and assurance 35
14 Public and patient involvement 36
15 Ethical considerations 38
16 Risk Management 38
17 Financing 39
18 Insurance 39
19 Publication and Dissemination 39
19.1 Publication 39
19.2 Informing participants 40
20 Disclosure of Interests 40
21 Figures and Tables 41

21.1 Figure 1: Schematic overview of study 41

21.2 Figure 2: Logic model 42

21.3 Figure 3: Progression criteria 43

21.4 Table 1: Study time/event matrix 44

22 Appendix 1 Information with regards to Safety Reporting in Non-CTIMP Research 47

23 References 49

3 List of Abbreviations and Definitions

AE	Adverse Event
CI	Chief Investigator
CRF	Case Report Form
ISRCTN	International Standard Randomised Controlled Trial Number
KCL	King’s College London
PI	Principal Investigator
Participant	An individual who takes part in a clinical trial
REC	Research Ethics Committee
RGO	Research Governance Office
RUSAE	Related Unexpected Serious Adverse Events
SAE	Serious Adverse Event
TMG	Trial Management Group
TSDMC	Trial Steering and Data Monitoring Committee
USM	Urgent Safety Measure

4 Summary/Synopsis

Full Title	Outdoor Mobility After Hip Fracture: A Feasibility Randomized Controlled Trial
Short Title/Acronym	OUTDOOR
Protocol Version number and Date	2.0 10/11/2023
IRAS Number	329085
Trial registration details	Plan for registration on ISRCTN prior to protocol implementation
Chief Investigator	Emma Godfrey
Study co-ordinator	To be appointed (post advertised July 2023; CI coordinating in interim)
Sponsor name	King's College London
Co-Sponsor	NHS Norfolk and Waveney Integrated Care Board
Funder(s)	NIHR Research for Patient Benefit [NIHR204040]
Medical condition or disease under investigation	Hip fracture
Trial Design	Feasibility randomised controlled trial
Methodology	Feasibility randomised controlled trial with embedded qualitative study
Study Duration	20 months (14 months from the start of recruitment window to last participant completing all assessments)
Purpose of clinical trial	The overarching aim is to determine the feasibility of a trial design to assess the clinical- and cost-effectiveness of an intervention designed to enable recovery of outdoor mobility among adults after hip fracture.
Primary objective	The primary objective of this feasibility trial is to determine whether the intervention was delivered as intended considering the five domains of intervention fidelity (design, training, delivery, receipt, and enactment).
Secondary objective(s)	Secondary objectives include:<br>• acceptability of the intervention to participants and therapists<br>• barriers and enablers to intervention delivery<br>• count of eligible, recruited and retained participants<br>• acceptability, completeness, and descriptive comparison of outcome (including economic) data collection<br>• count of inadvertent unblinding of outcome assessors<br>• count of adverse and serious adverse events<br>• indicative sample size for a definitive trial
Number of Participants	76 (60 patient participants, 16 professional participants)
Endpoints	The end of the study is the date when the last participant has completed all assessments, data queries are resolved and database locked, which is anticipated to be month 17 from study start
Main Inclusion Criteria	Participants: Adults aged 60 years or more, admitted to hospital from (and planned discharge to) home, self-reported outdoor mobility in the three-months pre-fracture, surgically treated for hip fracture, able to consent and participate. Participants recruited under the above 'inclusion criteria' whose circumstances change (e.g., planned discharge to home but then discharged to nursing/residential care) will be withdrawn before randomisation. They will not be replaced.

	Professionals: Therapists involved in the intervention arm of the feasibility trial, and managers who oversee services involved in the delivery of the intervention within the feasibility trial.
Main Exclusion Criteria	Participants: Adults aged less than 60 years, admitted to hospital from (or planned discharge to) nursing/residential care, no self-reported outdoor mobility in the three-months pre-fracture, non-surgically treated for hip fracture, who are likely to require two or more persons to support mobility on discharge, and/or who are unable to consent or participate.<br><br>Professionals: Therapists not involved in the intervention arm of the feasibility trial, and managers who do not oversee services involved in the delivery of the intervention within the feasibility trial.
Study Duration	20 months
Intervention	Both groups will receive usual care. Participants allocated to the intervention will also 1) receive a goal-orientated outdoor mobility programme with up to six therapist home visits and four telephone/MS TEAMS sessions to support; 2) support to engage with community activities and social enterprise groups; 3) feedback and motivation for unsupervised mobility training; and 4) access to a video of older adults sharing their recovery experience after hip fracture.
Statistical Methodology and Analysis	<u>Quantitative</u><br>A CONSORT flow diagram will display eligibility, recruitment, consent and follow-up rates. Confidence intervals for recruitment and retention rates will be produced. Completion rates will be estimated for outcome measures at each time-point, including resource use data. Patient-reported outcomes and acceptability will be summarised by allocated group at each follow-up, with descriptive statistics (measures of central tendency and dispersion). Between-group differences, including in changes from baseline, will be reported for the patient-reported outcomes with corresponding confidence intervals.<br><br><u>Qualitative</u><br>Data transcribed verbatim from semi-structured interviews and focus groups will be analysed using a thematic analysis approach. A random sample of intervention audio recordings will be sampled against intervention components to assess fidelity.
Data to be collected & associated storage arrangements	• Quantitative data (personal and pseudo-anonymised) stored in REDCap Academic, managed by Exeter CTU<br>• Qualitative data (pseudo-anonymised) stored on KCL SharePoint<br>• Paper based data stored at trial sites

Version Control

**Date:**	**Previous protocol amendments:**
10/11/2023	Addition of Section 9.5.7

5 Introduction

5.1 The problem

UK hospitals admit around 70,000 older adults with hip fracture annually.[1] The injury has been dubbed the "hip attack", due to its clinical severity and adverse consequences.[2] Even with surgery, there is a 5- to 8-fold increased risk for all-cause mortality in the first three months after hip fracture.[3] Among survivors, there are reported high rates of transition from independent living to nursing homes among persons with hip fracture.[4] These poor outcomes led 81 global societies to endorse a call to action for ongoing post-acute care of people whose ability to function is impaired by hip fractures.[5]

The response to the call to action should aim to improve outcomes that matter most to patients after hip fracture. We synthesised the evidence from 14 studies which explored 279 patient perspectives of recovery after hip fracture.[6] Across studies, patients considered recovery as a return to prefracture activities often requiring outdoor mobility e.g., gardening, shopping, participating in social events such as meeting friends, attending the theatre, and volunteering.[6] These priorities reflect the World Health Organization's definition of functional ability as 'all the health-related attributes that enable people to be and to do what they have reason to value'.[7]

"I just miss getting up and getting out. I never stayed in. I'd go out in the morning and come back and then I'd go out again." (92-year-old female 5 weeks after hip fracture surgery) [8]

"I'm very careful now, almost excessively so. . .. I'm careful when I'm out walking . . . Then I take it really easy! Look down at the ground . . . Now I'm afraid that it will happen again. And [that I will] break something else". (66-year-old female in the first month after discharge from hip fracture surgery) [9]

In work funded by our previous RfPB award (PB-PG-1216-20031: Sheehan PI), we reported that 74% of patients had outdoor mobility pre-fracture, but only 9% of these individuals recovered this mobility by 30 days post-fracture,[10] increasing to 26% by 120 days post-fracture.[11] Despite this low level of return to pre-fracture outdoor mobility, research and clinical guidelines focus on acute rehabilitation after hip fracture, with community rehabilitation limited to in-home mobility.[12-14] There is little consideration of interventions to improve outdoor mobility as evidenced by a national survey of community rehabilitation after hip fracture.[15] Rehabilitation explicitly targeting outdoor mobility is therefore needed to enable older adults to recover activities which they value most after hip fracture.

5.2 Important of research to patients and health care services

Patients identified the goal that matters most for them which is regaining the ability to partake in pre-fracture activities, often outside the home.[6] From national audit data, 74% of patients were able to mobilise outdoors before their hip fracture.[10] Of these, 91% were admitted from and discharged to their own home.[10] However, currently only one in four patients recover their ability to go outdoors by four months after hip fracture.[11]

Optimising recovery of outdoor mobility for older adults after hip fracture has the potential to significantly improve the quality of life of patients and their caregivers. Attributing improvements in patients' quality of life to outdoor mobility is based on increased opportunities for physical activity, promoting independence[16] whilst negating the risks of comorbid disease and illness,[17] and social isolation/loneliness.[18] For caregivers, their quality of life may be improved by reducing the need to adapt to changes in relationships and dependency.[19]

Deterioration in mobility is associated with increased risk of transition to a care home.[20 21] The proportion of patients residing in a UK care home increased by 25% from the time of hip fracture to one-year post-fracture.[22] Mobility loss after hip fracture is also associated with subsequent falls and fragility fractures.[23] These adverse consequences are key drivers of the estimated £1.1 billion annual UK hospital cost associated with incident hip fractures.[22] Improving outdoor mobility therefore has the potential to reduce the burden posed by hip fracture on health and social services.

To address this challenge, we designed an intervention to support older adults to recover their outdoor mobility and reduce burden on health and social services. Our intervention, based on theory[24-26] and building on previous research[27], was refined during two intervention development workshops with 20 international stakeholders including public and patient involvement representatives (further detail in Research Plan, Intervention). The intervention was designed for implementation in the NHS, taking cognisance of resource availability.

5.3 Previous evidence

5.3.1 Guidelines and research priorities

Current clinical guidelines provide limited recommendations for community rehabilitation after hip fracture. The National Institute for Health and Care Excellence (NICE) guidance for hip fracture recommends early supported discharge from hospital.[12] The Chartered Society of Physiotherapy recommends review by a rehabilitation provider within 72 hours of hospital discharge and sharing of assessments and plans across the multidisciplinary team.[13] The 2021 American Physical Therapy Association Clinical Practice Guideline recommends functional and gait/mobility training.[14] None include guidance for interventions aimed at achieving outdoor mobility. The **James Lind Alliance** identified this absence of evidence as a top 10 priority for lower limb fragility fracture among older adults:[28]

James Lind Alliance Top 10 Priority: "What is the best physiotherapy and/or occupational therapy regime for adults during out-of-hospital recovery from a fragility fracture of the lower limb?"

5.3.2 Systematic reviews of outdoor mobility interventions

We completed a series of systematic reviews which sought to determine the effectiveness of community-based rehabilitation interventions which incorporate outdoor mobility on outdoor mobility, physical activity, endurance, and falls-related self-efficacy among 1) older adults with hip fracture,[27] and 2) older adults more broadly.[29]

For the hip fracture review, we identified 11 randomised controlled trials (RCTs) which included an outdoor mobility component in their rehabilitation intervention.[27] There were methodological concerns related to unblinded outcome assessors and a lack of precision in observed effect size estimates across included trials. There was a suggestion (confidence interval crosses null) of a small effect on outdoor mobility (two trials with 285 participants, risk difference 0.19; 95% confidence interval (CI): -0.21 to 0.58) and falls-related self-efficacy (three trials with 363 participants, standardised mean difference 0.25; 95% CI: -0.29 to 0.78). The aerobic training arm of the feasibility trial by Mangione et al was the only intervention to achieve a clinically meaningful (but not statistically significant) between-group difference for endurance at the end of the intervention.[30] Five trials assessed the effect of their intervention on health-related quality of life as a secondary outcome reporting between group/no between group differences and significance levels but no crude data to enable meta analysis.[30-34] Two of the five trials reported an effect on health-related quality of life at intervention end,[30 33] and 12-month follow-up.[33]

Half of the trials included in the hip fracture review incorporated a psychological treatment component (goal setting and/or motivation). Most trials did not provide sufficient detail to replicate the intended outdoor mobility component, nor did they provide detail on the extent to which the outdoor mobility component was actually achieved. Potential benefits in falls-related self-efficacy, outdoor mobility, and/or health-related quality of life were observed for interventions where outdoor mobility was a more central treatment component. The intervention by Ziden et al. focused explicitly on increasing outdoor mobility through physical activity and cognitive behavioural interventions.[35] The aerobic training arm of the trial by Mangione et al focused on 20 minutes of indoor and outdoor walking.[30] While the results of both these studies showed some promise, Ziden (Sweden)[35] failed to blind outcome assessors to group allocation, while Mangione (USA),[30] was a small feasibility trial (n=22) and so not statistically powered. No relevant trials were identified from searches of trial registries.

For the review of older adults more broadly, we identified 28 randomised controlled trials which included an outdoor mobility component in their rehabilitation intervention.[29] The quality of included studies was moderate to low. Analyses were stratified by target population (proactive [community-dwelling n = 10] or reactive [with illness/injury n = 18]).[29] Rehabilitation interventions for reactive populations significantly increased physical activity (seven trials with 587 participants, Hedge's g 1.32; 95% CI: 0.31 to 2.32), outdoor mobility (two RCTs with 663 participants, Getting out of the house as much as wanted (rate ratio 1.74, 95% CI 1.24 to 2.44 at 10-months), likelihood of making a journey (rate ratio 1.42, 95% CI 1.14 to 1.67 at 6-months)), and endurance (four trials with 392 participants, Hedges g 0.24; 95% CI: 0.04 to 0.44) at intervention end versus usual care. Results were also suggestive (confidence interval crosses null) of a small effect on falls-related self-efficacy (four RCTs with 429 participants, Hedge's g 0.27; 95% CI: -0.18 to 0.71). Observed effects were preserved for physical activity and falls-related self-efficacy but not endurance at final follow-up (up to 12-months). Eight trials assessed the effect of their intervention on health-related quality of life. In addition to the hip fracture trials outlined above, two trials reported an increase in health-related quality of life for intervention (but not control) participants with chronic obstructive pulmonary disease,[36 37] while two trials reported no effect on health-related quality of life for older adults at risk of falls[38] and those after stroke.[39]

Most trials operationalised their outdoor mobility intervention component as a walking programme supported by behaviour change techniques. Few included references to assistive devices (walking aids, scooters) or transport. For the UK trials, by Logan and colleagues, the intervention targeted a broader definition of outdoor mobility which included walking, use of assistive devices, resuming driving, and taking a taxi or public transport.[39 40] Participants were supported by up to 7 [40] or 12 [39] sessions with a therapist to build confidence during practice of outdoor mobility. Participants in the intervention groups took more outdoor journeys[40] and were more likely to make an outdoor journey[39] at intervention end and 10-12-month follow-up compared with the control group. Whilst this demonstrates potential value of these outdoor mobility targeted interventions they were evaluated among older adults post-stroke who may face different physical, psychological, and cognitive barriers to outdoor mobility compared with patient after hip fracture.[41]

We searched clinical trial registries on 09/03/2022 and noted no registered planned/ongoing trials which address outdoor mobility after hip fracture surgery. There is one community based RCT of enhanced rehabilitation after hip fracture currently underway in the UK.[42] While both interventions target a similar patient population in the community, the interventions are distinctly different. The funded trial does not focus on outdoor rehabilitation or societal participation.[42]

5.3.3 Summary

There is no clinical guidance for interventions aimed at achieving outdoor mobility after hip fracture. Results from two systematic reviews suggest a potential benefit of an outdoor mobility program which includes 1) walking, use of assistive devices and transport; and 2) a behaviour change component, with promise identified for components which relate to goal setting and motivation, on outdoor mobility, physical activity and endurance. [27 29] The systematic review also found a suggestion of a potential benefit with respect to falls-related self-efficacy (confidence intervals crossed null values for both reviews). [27 29] No trial identified in either review included an intervention component targeting anxiety related to outdoor mobility or fear of falling. This is despite the reported negative association between fear of falling and outdoor mobility behaviour.[43 44] Given the current high proportion of hip fracture patients who do not regain outdoor mobility, further research is required. Our new, theoretically informed intervention has the potential to help with this challenge.

6 Trial objectives and purpose

6.1 Research hypothesis

The overarching aim of this programme of work is to determine the feasibility of a trial design which is aimed to assess the clinical- and cost-effectiveness of an intervention designed to enable recovery of outdoor mobility among adults after hip fracture. Several uncertainties will be addressed first through a non-commercial randomised feasibility trial.

The null hypothesis states that it is not possible for the NHS to deliver a new outdoor mobility intervention for older adults who break their hip. The alternative hypothesis states that it is possible for the NHS to deliver a new outdoor mobility intervention for older adults who break their hip. If

shown to be feasible, a full-trial will be planned to see whether the new outdoor mobility intervention is better, if it is possible to accept the alternative hypothesis at the end of the current study.

6.2 *Primary objectives*

The primary objective of this feasibility trial is to determine whether the intervention was delivered as intended considering the five domains of intervention fidelity (design, training, delivery, receipt, and enactment).

6.3 *Secondary objectives*

Secondary objectives include assessment of the:

- acceptability of the intervention to participants and therapists
- barriers and enablers to intervention delivery
- count of eligible, recruited and retained participants
- acceptability, completeness, and descriptive comparison of outcome (including economic) data collection
- count of inadvertent unblinding of outcome assessors
- count of adverse and serious adverse events
- indicative sample size for a definitive trial

7 Trial design & Flowchart

7.1 *Trial Design*

A multi-centre pragmatic parallel group (allocation ratio 1:1) randomised controlled assessor-blinded feasibility trial.

A schematic overview of the study is provided by Figure 1. A time/event matrix of trial procedures and stages is outlined in Table 1.

Both groups will receive usual care. In addition, the intervention group will receive a new outdoor mobility intervention. The intervention will start around 30 days after a participant returns home and end when their mobility end goal is achieved, six visits have occurred, or 12 weeks have passed – whichever comes first. This time frame/duration was selected as an appropriate window to target outdoor mobility based on our previous analysis of audit data,[11] systematic reviews,[27 29] and public and patient involvement focus groups. We aim to follow up all participants at 12 weeks post-randomisation. We will also follow-up participants to 6-months post-randomisation if the timing of randomisation permits this follow-up within the trial data collection window (to assess feasibility of longer-term follow-up within the current funding timeframe and budget).

The primary objective of this study (to determine whether the intervention can be delivered as intended considering the five domains of intervention fidelity) will be met by analysis of data collected through completed treatment logs and audio recordings during intervention delivery.

Secondary objectives of the study will be met by analysis of screening, approach, consent, and completion logs (secondary objective 3), descriptive statistical analysis of outcomes assessed at 6-weeks, 12-weeks, and 6-months post-randomisation (secondary objectives 1,4,5,7), and qualitative

interviews of patients and community team leaders and service managers and focus groups of therapists at intervention end (secondary objectives 1,2,3). The secondary objective to determine the count of AE and SAE will be met through reporting procedures in place from the point of randomisation to 12-week follow-up (secondary objective 6).

7.2 Study Setting

Screening and consent will take place in the hospital setting. The intervention and usual care will start after a participant returns home. The intervention and usual care will be delivered by physiotherapists/occupational therapists/therapy assistants (hereafter referred to as 'therapists') at participating sites. We selected sites across the UK to ensure feasibility is assessed in a diverse range of contexts and local populations.

8 Participant selection

The target population is older adults surgically treated following a hip fracture who were able to mobilise outdoors from their home pre-fracture and planned discharge to home. A member of the direct care team will screen potential participants admitted for hip fracture surgery for eligibility in participating hospitals.

We previously reported on National Hip Fracture Database data for adults aged 60 years or more surgically treated for hip fracture in England and Wales that 74% of patients had outdoor mobility pre-fracture.[10] Extrapolating this to the 63,284 patients in 2020 National Hip Fracture Database equates to 46,830 patients across 173 hospitals, or 271 patients per hospital per year. We anticipate 32% of those eligible who are approached will consent to participate, from previously observed recruitment rates for the same population for a community-based intervention.[42]

8.1 Participant inclusion criteria

Adults aged 60 years or more, admitted to hospital from (and planned discharge to) home, with outdoor mobility in the three-months pre-fracture, surgically treated for hip fracture, able to consent and participate.

- Aged 60 years or more
- Admitted to hospital from (and planned discharge to) home
- Self-reported outdoor mobility in the three-months pre-fracture
- Surgically treated for hip fracture
- Able to consent and participate

8.2 Participant exclusion criteria

- Aged less than 60 years
- Admitted to hospital from (or planned discharge to) nursing/residential care
- No self-reported outdoor mobility in the three-months pre-fracture

- Non-surgically treated for hip fracture
- Who are likely to require two or more persons to support mobility on discharge
- Unable to consent or participate

Justification for exclusions:

1) aged less than 60 years, to align with the National Hip Fracture Databases definition of the target population.[1]
2) admitted from (or planned discharge to) residential/nursing care, as the safety profile of the intervention would vary to those admitted from home (e.g., prescription of unsupervised intervention components may not be possible).
3) without outdoor mobility in the three-months pre-fracture, as the intervention seeks to support the recovery of prefracture outdoor mobility.
4) not surgically treated, as this treatment approach is reserved for around 2% of patients in the UK who are often at the end of life.
5) requiring two or more persons to support mobility on discharge, as the intervention would require visits by two therapists to support implementation (which on consultation with community therapists would not be feasible).
6) participants require capacity to consent due to the safety profile of the intervention (the intervention includes unsupervised outdoor mobility).

8.3 Professional eligibility criteria

- Therapists involved in the intervention arm of the feasibility trial, or
- Managers who oversee services involved in the delivery of the intervention within the feasibility trial.

9 Trial procedures

9.1 Participant recruitment

9.1.1 Participants

A member of the direct care team will screen potential participants for eligibility during the inpatient stay after hip fracture surgery, and determine the participants interest in learning more about the study. A suitably trained individual (clinician or trust research staff) will provide information leaflets and answer questions, obtain written consent (or not) to participate (or consent to contact on discharge home), and add to the screening log. Where possible, potential participants will be given at least 24 hours to consider their participation in the study. For participants approached on the day of discharge, on-the-day recruitment will be permitted should the suitably trained individual be assured that participants have understood the information and have had the opportunity to ask any questions. For participants who provide consent to contact on discharge home, they will be followed

up by the community teams to answer any questions and obtain written consent (or not) to participate, and added to the screening log. Reasons for ineligibility and declining to participate will be documented if they are offered without influence.

Participants recruited under the above 'inclusion criteria' whose circumstances change (e.g., planned discharge to home but then discharged to nursing/residential care) will be withdrawn before randomisation. They will not be replaced.

9.1.2 Professionals

Therapists who were involved in delivery of the intervention arm of the feasibility trial will be invited to take part in semi-structured focus groups on treatment acceptability and fidelity (inclusive of barriers and facilitators to implementation). A member of the research team will outline the aims, methods, benefits and potential harms of the qualitative study and provide a participation information leaflet during therapist training prior to the start of the study. During this training sessions, therapists will be asked to provide consent to contact from the research team towards the end of the study (their name and email address will be stored on an Excel Spreadsheet on secure KCL SharePoint server). Towards the end of the study a member of the research team will contact those who provided consent to contact and answer any questions prior to obtaining written informed consent to the interview study.

Managers of services involved in the delivery of the intervention within the feasibility trial will be invited to take part in semi-structured interviews on treatment acceptability and fidelity (inclusive of barriers and facilitators to implementation). A member of the research team will outline the aims, methods, benefits and potential harms of the qualitative study and provide a participation information leaflet via email. At least 24 hours later, a member of the research team will contact the manager answer any questions prior to seeking written informed consent to the interview study.

9.2 Screening Procedures

A screening log will document the number of adults admitted with hip fracture, the number screened (the number ineligible and reasons for ineligibility, and reasons for eligible but not screened), and the number who declined (and why). The screening log will be completed by the clinical team with a site principal investigator (PI) responsible for ensuring the accuracy of the log. Age, sex and ethnicity will be collected (and reported anonymously) for patients screened, but who do not subsequently provide consent, allowing generalisability of the randomised participants as well as the screened patients to be interpreted.

9.3 Randomisation Procedures

Participants will be allocated in 1:1 ratio to intervention or control groups. The Exeter Clinical Trials Unit (ExeCTU) will generate the random allocation sequence, stratified by recruiting site, with minimisation (with random element) for pre-fracture mobility (freely mobile outdoors without aids, mobile outdoors with one aid, mobility outdoors with two aids or frame).

Randomisation of a participant will take place after baseline data collection is completed following discharge from hospital, using a secure internet-based system, developed and maintained by ExeCTU

to ensure allocation concealment. Treatment allocation will be linked to a participant identification number (PID) and the clinical team will be notified. Group allocation will be documented in the patient's medical notes and on an enrolment log.

There are no anticipated circumstances under which the randomisation codes may need to be broken.

9.4 Blinding

The group allocator and assessors of patient reported outcomes will be blind to group allocation. The analysing statistician will be blinded at least until the statistical analysis plan is drafted.

9.5 Intervention Procedures

9.5.1 Support from existing evidence

Results from two systematic reviews suggest a potential benefit of an outdoor mobility program which includes 1) walking, use of assistive devices and transport; and 2) a behaviour change component with promise identified for components which relate to goal setting and motivation, on outdoor mobility, physical activity and endurance.[27 29] There was also a suggestion of a potential benefit with respect to falls-related self-efficacy.[27 29] No trial identified in either review included an intervention component targeting anxiety related to outdoor mobility or concerns about falls. This is despite the reported negative association between concerns about falls and outdoor mobility behaviour.[43 44]

9.5.2 Theoretical frameworks

Building on the findings from the two systematic reviews, we employed Webber's theoretical framework for mobility in older adults which defines mobility as the ability to move oneself (by walking, with assistive devices or transport) within community environments that expand from a room within a person's home to outdoors - a person's garden or driveway, to their local neighborhood of nearby streets and parks, to the service community (banks, shops, GP office), and then the surrounding area and beyond.[24] The concept of mobility is portrayed through five fundamental determinants – physical, cognitive, psychosocial, environmental, and financial.[24] These determinants will hold different degrees of importance depending on the person, where a person is going and how, but the determinants are related to each other and become more complex the further a person travels from their own home.[24] This framework enables us to build on the previous evidence by considering mobility in the current proposed trial as walking, use of assistive devices and transport.

Operationalising Webber's determinants required consideration of two additional theories. First, the COM-B model of behaviour change targeting participants need for capability, opportunity, and motivation to generate and maintain a desired behaviour.[25] The previous evidence identified by the systematic reviews identified behaviour change as a key intervention component – more specifically goal setting and motivation. Second, Normalisation Process Theory which looks at how to embed a practice into 'work as usual' through 4 components. Coherence relates to understanding and making sense of a practice (here 'outdoor mobility'), cognitive participation – engaging and participating

with the practice, collective action – the joint 'work' needed to enact the practice, and reflexive monitoring – reflecting and appraising the practice over time to ensure it becomes routinely embedded.[26] This theory was considered essential for designing an intervention with future sustainability in mind.

We incorporated each of these three theories into the development of our programme theories and logic model for the proposed outdoor mobility intervention.

9.5.3 Development

In August and September 2021, we convened two remote intervention development workshops comprised of patients, carers, physiotherapists, triallists, implementation and behaviour change scientists, as well as a panel of experts in rehabilitation for patients after hip fracture from the UK, Denmark, Norway, Spain, USA, Canada, Brazil and Australia (n=20 participants). At the workshops, we presented the rationale and key question (*what intervention would enable older adults to take part in activities outside the home after hip fracture?*), data/results from existing evidence, including results of our two systematic reviews, outline of current UK provision, proposed theoretical frameworks, and draft programme theories and logic model. We then followed a nominal group technique inviting participants to generate intervention ideas silently prior to sharing in a round-robin format.[45] All ideas were documented. Participants sought and provided clarification on shared ideas. Top ideas were then prioritized for incorporation into the subsequent draft programme theories and logic model (Figure 2) which were finalised after the second workshop.[45]

9.5.4 Programme theories

1. For older adults who were mobile outdoors prior to hip fracture, health-related quality of life post-fracture is enhanced by regaining outdoor mobility;

2. Achievement of physical, psychosocial, and cognitive capability to go outdoors requires tailored, structured, and graded support with mechanisms to monitor and preserve gains.

9.5.5 Intervention Description

The intervention will start around 30 days after a participant returns home and end when their mobility end goal (patient and therapist shared goal) is achieved, or six visits have occurred, or 12 weeks have passed – whichever comes first. It will be delivered by therapists (physiotherapists/ occupational therapists/ therapy assistants). Online intervention training will be provided to treating therapists prior to delivery. To enable fidelity assessment, therapists will audio record intervention sessions, with participants' consent. Audio recordings will be supplemented by study-specific 'intervention' questionnaires completed by the physiotherapist/occupational therapist/therapy assistant after each session with a participant.

Intervention participants will receive usual care (*see 9.5.6*) and:

9.5.5.1 Motivation – social support

Participants will be provided access (via therapist held tablet) to a video of older adults who incurred hip fracture sharing their experience of recovery. Therapists will also provide participants with a piece of paper with a Kings College London SharePoint server website link written on it, should

participants wish to watch the video again later in their own time. The content of the video includes discussion by four older adults about their *capability*, *opportunity*, and *motivation* to generate and maintain their desired behaviour of outdoor mobility (including walking, taking public transport) after hip fracture. More specifically, the video targets motivation through the behaviour change techniques *opportunity for social comparison* and *social support (practical, general, and emotional)*.

9.5.5.2 Goal-orientated mobility programme

Participants will have a telephone call/MS TEAMS call with a therapist to set an outdoor mobility programme goal. This goal will be meaningful to the participant and be deemed achievable within the scope of the programme by the therapist.

The 'programme goal' will be broken down into a maximum of four 'intermediate goals' by the therapist. These intermediate goals will promote movement through life spaces increasingly further from a participant's home and towards their programme goal. These goals should be individually tailored to account for a participants prefracture abilities.

An example of a programme goal and related intermediate goals is specified in Box 1.

Box 1: Example goals	
Programme goal	Travel on public transport (10-minute walk to bus stop, 20-minute bus journey, 5-minute walk to restaurant) to meet family for lunch, return by car with family.
Intermediate goals ↑	Walk to bus stop with the use of one crutch/stick, take bus to town and back independently.
	Walk to bus stop with the use of one crutch/stick, take bus to town and back with another person.
	Walk continuously for 10-minutes outside with the use of one crutch/stick independently.
	Walk continuously for 10-minutes outside with the use of two crutches with another person.

The programme will be supported by a maximum of six therapist home visits (target mean of four) to enable a treating therapist to provide supervised support for practice and progression of intermediate goals. Therapists will employ motivational interviewing (engage, focus, evoke and plan) during supervised sessions (see 9.5.5.5).[46]

The participant will be asked to practice independently and/or with family/friends between therapist visits. They will be asked to document this practice in a diary (whether completed or not, independently or with another person, how felt during and after).

The goal-orientated mobility programme targets behaviour change techniques related to capability

(*commitment*, *behavioural contract*), opportunity (*action planning, habit formation*), and motivation (*goal setting*) for outdoor mobility at increasing distances from home.

9.5.5.3 Motivation – additional professional support

In between therapist home visits, the participant will be supported by up to four telephone/MS TEAMS calls. These calls will be structured around the participant diary and look to reinforce motivational interviewing strategies employed during supervised sessions. This additional support targets motivation through behaviour change techniques related to *feedback on behaviour*, *others monitoring with awareness*, and *self-monitoring* of both the behaviour and outcome of the behaviour.

9.5.5.4 Transition to independence

During home visits and/or telephone calls, therapists will plan for ongoing recovery after the goal-orientated mobility programme ends. This planning will include supporting participants to engage with their local community activities and social enterprise groups. The availability of activities and groups will vary according to the participants place of residence. The activities and groups of interest will be determined by the participant depending on their personal preferences, and should be reinforced by the therapist. This transition to independence targets motivation through behaviour change techniques related to *social support*, *action planning*, *verbal persuasion to boost self-efficacy,* and *restructuring of the social environment*.

9.5.5.5 Additional therapist training

As part of the intervention, therapists will receive training in motivational interviewing.[46] Motivational interviewing will equip therapists with the strategies to reinforce engagement, focus on the change required to achieve goals and whether this is clear to the participant or not, evoke motivation for change by e.g., shifting to a greater stage of readiness (“I wish” to “I will”), and plan considering where, when how and with whom.

In particular, the training will focus on strategies for supporting older adults with concerns about falls related to outdoor mobility. We conducted a systematic review (under review at journal) of factors prognostic of concerns about falls following hip fracture. We identified factors amenable to change which therapists can target through motivational interviewing. These factors included fatigue, safe mobility, consequences of not moving, encouragement and feedback, locus of control, self-confidence, and worries about future and past. The training will support therapists to align these factors to the COM-B model of behaviour change and appropriate behaviour change techniques that can be employed through motivational interviewing. Therapists will observe motivational interviewing in practice, practice motivational interviewing themselves, and be provided with access to a recording of the training after the session. They will be offered a revision training session that is therapist-led to discuss any concerns they have in putting motivational interviewing into practice.

9.5.6 Usual care

The intervention and comparator group will both receive usual care. A survey of UK community rehabilitation for older adults after hip fracture identified two models for those discharged home:

early supported discharge with community rehabilitation for four to six weeks or, discharged home under GP care with referral to community services as needed.[15] Where provided, physiotherapy focused on strengthening exercises (100%), progressive resistance training (91%), weight-bearing exercises (95%), gait training (79%), exercise sheets (91%), encouragement of walking and climbing stairs (98%), and transferring (91%) (mode 30 minutes weekly/fortnightly for 4-6 weeks). Occupational therapy focused on transfer assessment, activities of daily living (e.g., grooming/personal hygiene, dressing, toileting/continence, transferring, and eating), home environment and social support (mode 60 minutes weekly/fortnightly for four to six weeks). Outdoor mobility and behaviour change techniques[25] were not included in routine community rehabilitation.

To enable fidelity assessment (and to monitor for contamination between trial intervention and usual care arms), therapists will complete a study-specific 'usual care' questionnaire after each supervised session of usual care.

9.5.7 Outdoor intervention -example of schedule

• **Telephone call 1**

a. Set outdoor mobility programme goal using motivational interviewing (MI) strategies and behaviour change techniques (BCTs) (e.g. problem solving, action planning, information about health consequences, pros and cons, verbal persuasion about capability). The goal should require outdoor mobility, be meaningful to the patient, and achievable within the scope of the programme.
b. Determine (logistically) what will be required to complete the outdoor mobility goal e.g. if the goal is to go for coffee with a friend how would they normally get there? Walk to the bus, travel ten stops, short walk to the café, then return. This will allow creation of intermediate goals.
c. Schedule first home visit.

•**Home visit 1**

a. Show video of older adults discussing their experiences of recovery. Provide piece of paper with link to video to watch again later.
b. Outline intermediate goals in pursuit of the programme goal.
c. Practice first intermediate goal while using MI strategies and BCTs (feedback and monitoring, framing/reframing, verbal persuasion about capability).
d. Set plan for practice between home visits with social support as available (BCT – social support, restructuring the social environment, habit formation).
e. Provide and describe how to complete home diary.
f. Schedule next home visit.

• **Home visit 2**

a. Discuss home diary – extent of practice, how felt during and after (MI strategies and BCTs e.g. feedback and monitoring, framing/reframing, verbal persuasion about capability).

b. Practice intermediate goal while using MI strategies and BCTs (feedback and monitoring, framing/reframing, verbal persuasion about capability).
c. Progress to next intermediate goal as deemed appropriate.
d. Set plan for practice between home visits with social support as available (BCT – focus on past success, social support, restructuring the social environment, habit formation).
e. Schedule next telephone call and home visit.

- **Telephone call 2**

a. Discuss home diary – extent of practice, how felt during and after (MI strategies and BCTs e.g. feedback and monitoring, framing/reframing, verbal persuasion about capability).
b. Remind of home visit plan.

- **Home visit 3**

a. Practice intermediate goal while using MI strategies and BCTs (feedback and monitoring, framing/reframing, verbal persuasion about capability).
b. Progress to next intermediate goal as deemed appropriate.
c. Set plan for practice between home visits with social support as available (BCT – focus on past success, social support, restructuring the social environment, habit formation).
d. Review progress towards programme goal.
e. Schedule next home visit.

- **Home visit 4**

a. Discuss home diary – extent of practice, how felt during and after (MI strategies and BCTs e.g. feedback and monitoring, framing/reframing, verbal persuasion about capability).
b. Practice intermediate goal while using MI strategies and BCTs (feedback and monitoring, framing/reframing, verbal persuasion about capability).
c. Progress to next intermediate goal as deemed appropriate.
d. Review progress towards programme goal.
e. Set plan for practice between home visits with social support as available (BCT – focus on past success, social support, restructuring the social environment, habit formation).
f. Schedule next telephone call and home visit.

- **Telephone call 3**

a. Discuss home diary – extent of practice, how felt during and after (MI strategies and BCTs e.g. feedback and monitoring, framing/reframing, verbal persuasion about capability).
b. Discuss opportunities for community engagement e.g. previous activities or local community activities available through social enterprise groups. This should be guided by the participants interest and activities available in the local area (BCTs – action planning, social support, restructuring the social environment, verbal persuasion about capability).
c. Remind of home visit plan.

- **Home visit 5**

a. Discuss home diary – extent of practice, how felt during and after (MI strategies and BCTs e.g. feedback and monitoring, framing/reframing, verbal persuasion about capability).

b. Practice intermediate goal while using MI strategies and BCTs (feedback and monitoring, framing/reframing, verbal persuasion about capability).
c. Progress to next intermediate goal as deemed appropriate.
d. Set plan for practice between home visits with social support as available (BCT – focus on past success, social support, restructuring the social environment, habit formation).
e. Schedule next telephone call and final home visit.

• **Telephone call 4**

a. Discuss home diary – extent of practice, how felt during and after (MI strategies and BCTs e.g. feedback and monitoring, framing/reframing, verbal persuasion about capability).
b. Discuss opportunities for community engagement e.g. previous activities or local community activities available through social enterprise groups. This should be guided by the participants interest and activities available in the local area (BCTs – action planning, social support, restructuring the social environment, verbal persuasion about capability).
c. Remind of final home visit plan.

• **Home visit 6**

a. Discuss home diary – extent of practice, how felt during and after (MI strategies and BCTs e.g. feedback and monitoring, framing/reframing, verbal persuasion about capability).
b. Complete programme goal while using MI strategies and BCTs (feedback and monitoring, verbal persuasion about capability, habit formation).
c. Set plan for practice going forward with social support as available (BCT – focus on past success, verbal persuasion about capability, social support, restructuring the social environment, habit formation).

9.6 Schedule of Assessments for each visit

Participants will undergo screening, consent, baseline assessment, assessment at 6- and 12-weeks post-randomisation. We will also complete an assessment at 6 months post-randomisation if the timing of randomisation permits this follow-up within the trial data collection window. All assessments will be completed over the telephone or via MS TEAMS.

Screening (in hospital): Confirmation aged 60 years or more, admitted to hospital from (and planned discharge to) home, with self-reported outdoor mobility in the three-months pre-fracture, surgically treated for hip fracture, able to consent and participate, age, sex, and ethnicity.

Consent (in hospital/at home): Written informed consent to participate (/contact on discharge) will be completed in hospital. For participants who provide consent to contact in hospital, they will be followed up by the community teams to obtain written consent to participate. For those who provide consent/consent-to-contact, fracture type, surgery type, Abbreviated Mental Test and the Clinical Frailty Scale will be collected.

Baseline (at home): Following consent, and prior to randomisation, a member of the research team will collect participant characteristics (see 21.4). They will also collect patient-reported outcome measures which satisfy the core outcome set for hip fracture trials[47]:

a) death (online death records, screened prior to attempt to contact participant)
b) health-related quality of life (EuroQoL EQ-5D-5L[48])
c) community mobility (University of Alabama Life Space Assessment[49])
d) activities of daily living (Nottingham Extended Activities of Daily Living[50])
e) falls related self-efficacy (Falls Efficacy Scale-International[51])
f) pain (Numeric Rating Scale[52])
g) bespoke resource use data collection form (including health and social care, informal care and paid/unpaid work)

6-weeks post randomisation (at home): At 6-weeks post randomisation, a member of the research team will collect the following:

a) death (online death records, screened prior to attempt to contact participant)
b) health-related quality of life (EuroQoL EQ-5D-5L[48])
c) community mobility (University of Alabama Life Space Assessment[49])
d) activities of daily living (Nottingham Extended Activities of Daily Living[50])
e) falls related self-efficacy (Falls Efficacy Scale-International[51])
f) pain (Numeric Rating Scale[52])
g) exercise adherence (Exercise Adherence Rating Scale).
h) bespoke resource use data collection form (including health and social care, informal care and paid/unpaid work)
i) hospital readmissions (and reason for readmission)
j) adverse/serious adverse events (see 11.1)

12-weeks post randomisation (at home): At 12-weeks post randomisation, a member of the research team will collect the following:

a) death (online death records, screened prior to attempt to contact participant)
b) health-related quality of life (EuroQoL EQ-5D-5L[48])
c) community mobility (University of Alabama Life Space Assessment[49])
d) activities of daily living (Nottingham Extended Activities of Daily Living[50])
e) falls related self-efficacy (Falls Efficacy Scale-International[51])
f) pain (Numeric Rating Scale[52])
g) bespoke resource use data collection form (including health and social care, informal care and paid/unpaid work)
h) exercise adherence (Exercise Adherence Rating Scale).
i) adverse/serious adverse events (see 11.1)
j) hospital readmissions (and reason for readmission)
k) acceptability (brief validated questionnaire)[53]

For intervention participants only, a member of the research team will collect whether the participant engaged family/friends (yes/no) to support them with aspects of the intervention. In addition, the research team will purposively sample participants from different sites with different pre-fracture mobility, living status, and accommodation type for additional telephone/MS TEAMS

semi-structured interviews focused on intervention fidelity (inclusive of barriers and facilitators to implementation). Data collection will continue until no new themes emerge (saturation) or 50% of participants have been interviewed.[54]

6-months post randomisation (at home): At 6-months post randomisation, a member of the research team will collect the following (if the timing of randomisation permits this follow-up within the trial data collection window):

a) death (online death records, screened prior to attempt to contact participant)
b) health-related quality of life (EuroQoL EQ-5D-5L[48])
c) community mobility (University of Alabama Life Space Assessment[49])
d) activities of daily living (Nottingham Extended Activities of Daily Living[50])
e) falls related self-efficacy (Falls Efficacy Scale-International[51])
f) pain (Numeric Rating Scale[52])
g) bespoke resource use data collection form (including health and social care, informal care and paid/unpaid work)
h) adverse/serious adverse events (see 11.1)
i) hospital readmissions (and reason for readmission)
j) death (online death records, screened prior to attempt to contact participant)

Participants who are non-English language speakers will be supported to complete assessments (except patient reported outcome measures) with the use of local interpreting services. Patient-reported outcome measures which have an established translated, validated, and (where applicable) culturally adapted version in the appropriate language will be circulated by post to the participant with a pre-paid envelope for return direct to the research team.

9.7 Follow up Procedures.

Outcomes will be collected by telephone/MS TEAMS at six- and 12-weeks post-randomisation; we will also collect outcomes at six months post-randomisation if the timing of randomisation permits 6-month follow-up within the trial data collection window.

9.8 Modifications or discontinuation of intervention

Should the research team/Trial Steering and Data Monitoring Committee (TSDMC) deem a need for discontinuation of the trial (e.g., for urgent safety measures), the CI will report the early discontinuation via an NRES End of Study Declaration form to the main REC with a copy to the co-Sponsors.

9.9 Withdrawal / dropout of participants

Participants are free to withdraw from the trial at any point or a participant can be withdrawn by the CI/site PI. No sanctions will follow if the participant decides to leave the research at any time.

Participants who wish to withdraw will have the option of withdrawal from:

- All aspects of the trial but continued use of data collected up to that point.
- All aspects of the intervention but continued use of data collected up to that point and continue with planned completion of questionnaires and interviews as well as collection of information from routine health records for the purpose of primary and some secondary outcomes.
- Any ongoing aspects of the trial that require patient contact or completion of questionnaires, but permission to collection of information from routine health records for the purpose of primary and some secondary outcomes.

Participants can be withdrawn by the CI/site PI following:

- A change in the health status of the participant that the clinical team report justifies withdrawal (including admissions to the inpatient setting).

If withdrawal occurs, the primary reason for withdrawal (as available) will be documented in the participant's case report form (CRF). Participants who withdraw will not be replaced.

9.10 End of Trial Definition

The end of the study is the date when the last participant has completed all assessments, data queries are resolved and database locked, which is anticipated to be by month 17 from the study start.

10 Regulatory Approvals

The study protocol and other relevant documentation will be submitted to, and approved by, a REC, the HRA and the appropriate local R&D departments for each site prior to entering participants into the trial.

All correspondence with the REC will be retained.

10.1 Amendments

For any amendment to the study, the Chief Investigator, in agreement with the Sponsor, will submit information to the relevant regulatory bodies for review. Substantial amendments that require review by the REC will not be implemented until that review has been completed with a favourable outcome, and other mechanisms are in place to implement at site. The Chief Investigator will work with the sites local site R&D departments as well as the study delivery team to confirm ongoing Capacity and Capability for the study.

10.2 End of Study Reporting

10.2.1 End of Study Declaration

The end of the study will be declared to the REC that gave a favourable opinion (as per the above Regulatory Approvals section) within 90 days of the study ending.

If the study is terminated early, the study will end on the date the Sponsor formally declares the study terminated in writing. The main NHS REC will be notified of early termination within 15 days of the Sponsor deciding to end the study.

10.2.2 End of Study Reporting

The end of the study report will be submitted to the REC that gave a favourable opinion (as per the above Regulatory Approvals section) within 12 months of the study ending.

11 Safety & Adverse Events Reporting

11.1 Assessment of Safety

Participant safety will be determined through the reporting of adverse events (AE) and serious adverse events (SAE). The period for AE/SAE reporting will be from randomisation until final follow-up (6-months if the timing of randomisation permits 6-month follow-up within the trial data collection window, 12-weeks otherwise).

An adverse event (AE) is defined as any untoward medical occurrence in a study participant, which does not necessarily have a causal relationship with the trial intervention. Adverse events that will be collected and reported in this trial are limited to:

1. an exacerbation of a pre-existing illness.
2. fall that does not require hospitalisation.
3. an increase in the frequency or intensity of a pre-existing episodic event or condition.
4. continuous persistent disease or a symptom present at baseline that worsens following administration of the trial intervention.

A serious adverse event (SAE) is defined as an untoward occurrence that:

1. results in death.
2. is life threatening (at the time of the event).
3. requires unplanned hospitalisation or prolongation of existing hospitalisation.
4. results in persistent or significant disability or incapacity.
5. Other 'important medical events' may also be considered serious if they jeopardise the participant or require an intervention to prevent one of the above consequences.
6. leads to a personal data breach.

NOTE: The term "life-threatening" in the definition of "serious" refers to an event in which the participant was at risk of death at the time of the event; it does not refer to an event which hypothetically might have caused death if it were more severe.

All deaths occurring from randomisation until final follow-up (6-months if the timing of randomisation permits 6-month follow-up within the trial data collection window, 12-weeks otherwise) or withdrawal from the study, irrespective of their relationship to the intervention, will be documented by a notification of death form and entered into the trial database within 24 hours of identifying the death. Cause of death will be recorded where available.

Related **E**xpected **S**erious **A**dverse **E**vents (RESAE). For the current intervention, an unplanned hospitalisation following an injurious fall may be an expected SAE and related to the intervention, given the intervention encourages people to increase their outdoor mobility.

Related **U**nexpected **S**erious **A**dverse **E**vents (RUSAE) - A related and unexpected SAE is an event which is related to the intervention; and 'unexpected' – that is, the type of event is not listed in the protocol as an expected occurrence.

Where AEs/SAEs occur, the team (direct care team delivering the intervention and control arms) will follow Good Clinical Practice Guidelines for the reporting to the medical team responsible for the participant's care and notify the Principal Investigator (PI). PIs at all sites will report all AEs/SAEs to the CI via the study electronic database (REDCap). The CI (unblind to allocation) will determine relatedness. The CI will then be responsible for reporting SAEs to KCL RGO.

All SAEs that are to be reported to the RGO will be signed and dated and completed by the CI. Where an RESAE occurs that does not require immediate reporting, this RESAE will be reported in the Annual Progress Report and copied to the RGO, alongside any AEs that occur that are not classified as 'serious'.

RUSAEs will be reported immediately upon knowledge of the event to the RGO and always within 24 hours. Reports of RUSAEs will be submitted to the Main NHS/ HSC REC within 15 days of the Chief Investigator becoming aware of the event, using the appropriate template. The form will be completed in typescript and signed by the Chief Investigator. The main REC will acknowledge receipt of safety reports within 30 days. A copy of the SAE notification and acknowledgement receipt will also be sent to the RGO.

In the instance of a personal data breach, the breach will be immediately reported to the CI, Sponsor's, and to the Data Protection Officer/Information Governance Department of the site that incurred the breach. The report will include full details as to the nature of the breach, an indication as to the volume of material involved, and the sensitivity of the breach (and any timeframes that apply), steps that have been taken to mitigate the risk (trying to retrieve the data asking third parties to delete information that was sent to them in error) to enable an assessment of the full risk/impact of the breach. The Sponsor will determine whether the breach meets the definition of a serious breach and warrants reporting to the regulators including the ICO https://ico.org.uk/for-organisations/report-a-breach/personal-data-breach-assessment/.

A joint Trial Steering and Data Monitoring Committee (TSDMC) will be informed of the number, nature and review outcomes for all serious adverse events and be asked to recommend any necessary actions.

The final report will detail the number (events and individuals) and nature of all events (AEs and SAEs) reported to members of each site and/or research team and will be submitted to the REC with the sponsor copied.

Information with regards to Safety Reporting in Non-CTIMP Research is further specified in Appendix 1.

11.2 Urgent Safety Measures

A decision to implement an urgent safety measure (USM) can be made by the Sponsor, CI, PI and/or TSDMC in the event of identifying an immediate risk to participant safety. USM identified shall take immediate effect and the event will be notified to the REC no later than 3 calendar days from the date the measures are taken. Any incident identified by a site that may result in an USM must be communicated to the CI immediately via the institutional study mailbox. If the CI and the Sponsor consider the USM to affect all participants, all PIs must be informed of the USM. A protocol amendment will be submitted to the HRA and REC at the earliest opportunity (and within three days) following implementation of the USM. If the USM requires a temporary halt to the study, this will be notified by an amendment.

12 Data

12.1 Data to be collected

Data collection, including who the data is to be collected by, from where, the data type and the time point for collection, is outlined in Table 1. Data collection at baseline, 6 weeks post-randomisation, 12-weeks post-randomisation, and 6-months post-randomisation (if the timing of randomisation permits this follow-up within the trial data collection window) will be between the participant and a member of the research team over the telephone or MS TEAMS.

The following participant characteristics will be collected to enable baseline comparison between allocated groups: age, sex, ethnicity, Abbreviated Mental Test (AMT), Clinical Frailty Scale,[55] living status (lives alone, with independent spouse, with dependent spouse, with family, with other), accommodation type (house/ground floor apartment with step free access, house/ground floor apartment with step access, 2nd floor or higher apartment with lift access, 2nd floor or higher with no lift access, sheltered accommodation), prefracture mobility (independent, with aid, with support of 1 person), fracture type, surgery type, length of stay (from admission to discharge from acute hospital), and any additional inpatient stay following discharge from hospital e.g. intermediate care. The acceptability, completeness, and descriptive comparison of patient reported outcome data collection will be assessed through collection of patient-reported outcome measures (via standardised tools) which satisfy the core outcome set for hip fracture trials.[47] Additional patient-reported outcome measures (via standardised tools) which capture putative mechanisms (community mobility, falls related self-efficacy) for an effect on health-related quality of life (as the proposed primary outcome for a subsequent definitive trial) will also be collected.

Additional quantitative outcomes captured will include mortality (online death record search) and self-reported hospital readmissions (with reason which may/may not be directly related to their hip fracture surgery) as key performance indicators of care after hip fracture surgery; resource use using a bespoke data collection form to assess feasibility of collecting health economic data while

minimising the burden on participants; exercise adherence during intervention delivery for the intervention arm, and participant and therapist acceptability with the use of a brief validated questionnaire at the end of the trial. For those in the intervention arm, we will capture whether participants engaged family/friends (yes/no).

For patients who do not speak English, patient-reported outcome measures which have an established translated, validated, and (where applicable) culturally adapted version in the appropriate language will be circulated by post to the participant with a pre-paid envelope for return. Patient characteristics and additional quantitative outcomes will be collected using local interpreting services.

To assess fidelity for the intervention group, we will audio record intervention sessions between each participant and their supporting physiotherapist/occupational therapist/therapy assistant including discussion of questions/responses recorded in participant diaries (which are to be completed to capture unsupervised outdoor mobility training between visits). The audio recording will enable assessment of the five intervention fidelity domains (design, training, delivery, receipt, and enactment) identified by the National Institute of Health Behaviour Change Consortium.[56] Fidelity assessment will be supplemented by study-specific questionnaires completed by the physiotherapist/occupational therapist/therapy assistant after each supervised session for both intervention and usual care groups.

Qualitative data on perceived barriers and facilitators to intervention delivery will be captured through purposively sampled (site, pre-fracture mobility, living status, accommodation type, sampled by the research team) remote (telephone/MS TEAMS) semi-structured interviews between a member of the research team and participants (until data saturation is reached). Therapists will contribute their perspectives on barriers and facilitators to intervention delivery in four 60-minute online focus groups via MS TEAMS facilitated by the research team. We will target recruitment of at least 80% of the therapists involved in intervention delivery at each site in each focus group. We will also extend invitations for interviews between a member of the research team and people at higher organisational levels (e.g., local allied health professional community team leaders and service managers) for their perspectives on potential barriers and facilitators.

Data collection forms (including topic guides) are detailed in additional files.

12.2 Data handling and record keeping

Data will be handled in adherence to the UK General Data Protection Regulation and Data Protection Act 2018.

12.2.1 Electronic database

Quantitative data, including participant demographics, will be entered into electronic case report forms (eCRFs) and managed in an instance of REDCap, an Electronic Data Capture system developed by Vanderbilt University. REDCap will be hosted securely within University of Exeter's Amazon Web Services (AWS) account. Data will be securely stored in AWS data centres in the UK with access

limited to only authorised staff. The REDCap database will be encrypted at rest. Access to REDCap public facing website via web browser will be encrypted using the appropriate Transport Layer Security (TLS) protocol.

Study projects and eCRFs will be built and validated in REDCap a priori to appropriate standards by ExeCTU in collaboration with the research team. Data collected in REDCap will be pseudo-anonymised with the use of the patient identification number as the primary identifier of the participant for data entered to REDCap. The eCRFs will be programmed to generate notifications for possible data entry errors (e.g. range checks) and missing key data points. Password protected user entry will be limited to therapists and members of the research team who need to enter or analyse data. Patient contact details will be available to 1) site therapists to arrange intervention delivery; and 2) authorized members of the research team who are required to contact participants for data collection/interviews/to send study findings at the end of the trial.

After completion of the trial, quantitative data will be transferred from Exeter CTU via a secure file sharing platform to KCL where it will be stored for 5 years after which it will be destroyed in line with KCL policies on data destruction. Following publication of the primary paper, anonymised electronic REDCap data will be exported and stored alongside anonymised transcriptions of interviews indefinitely on the King's Open Research Data System (https://www.kcl.ac.uk/researchsupport/managing/preserve), with proof of ethical approval as a condition of access.

12.2.2 Paper-based data

Paper-based identification, screening and enrolment logs, and consent forms, will be completed by therapists at participating sites. Paper-based study specific questionnaires will be completed by therapists delivering the intervention and comparator in the community. All paper-based data will be stored in a locked cabinet/drawer within a secure room at the participating site. Therapists will be required to enter all paper-based quantitative data to electronic CRFs on the REDCap Cloud database (see 13.2.1). Paper-based data (including consent forms) will be stored for 5 years after which they will be destroyed in line with local Trust policies on data destruction. For patients who do not speak English, pseudo-anonymised patient-reported outcome measures returned by post will be stored in a locked cabinet in a restricted access room and building at KCL for 5 years after which they will be destroyed in line with KCL policies on data destruction.

12.2.3 Qualitative data

For intervention group participants, all supervised treatment sessions will be audio-recorded to enable a direct assessment of treatment fidelity (primary objective). These audio-recordings will be uploaded by the therapist to a secure KCL SharePoint server accessible only to the research team, labeled with the participant identification number after which the recording will be deleted from the audio recording device.

For a sample of participants, the research team will audio-record semi-structured interviews completed at the end of their time in the trial. The research team will also audio-record semi-structured interviews with local allied health professional community team leaders and service

managers completed by MS TEAMS and focus groups with therapists via MS TEAMS. These audio-recordings will be uploaded by the research team to a secure KCL SharePoint server accessible only to the research team, pseudo-anonymised with the participant identification number/file identification number (for leaders, managers and focus groups) after which the recording will be deleted from the audio recording device. These recordings will be transcribed verbatim and anonymised after which the audio-files will be permanently deleted.

Following publication of the primary paper, anonymised transcriptions of interviews will be stored on the King's Open Research Data System indefinitely (https://www.kcl.ac.uk/researchsupport/managing/preserve), with proof of ethical approval as a condition of access.

12.3 Access to the dataset

Access to REDCap will be limited to site investigators and members of the research team who require access to enable intervention delivery and/or data entry, and to the Sponsor for monitoring and auditing purposes. The database will also be accessible to dedicated members of ExeCTU responsible for database management, export, and/or analysis. Access rights will be managed by specific role based access, ensuring staff can only perform functions within REDCap according to their role.

Access to qualitative data will be limited to members of the research team who require access for analysis and to the Sponsor for monitoring and auditing purposes. Further, access to audio-recordings of interviews and focus groups will be granted to an external transcription service 'The Typing Works' who will retain access only for as long as required to transcribe the recordings, after which access will be revoked. A service level agreement will be put in place between Kings College London and The Typing Works.

Following publication of the primary paper, anonymised electronic REDCap data will be exported and stored alongside anonymised transcriptions of interviews indefinitely on the King's Open Research Data System (https://www.kcl.ac.uk/researchsupport/managing/preserve), with proof of ethical approval as a condition of access.

13 Statistical considerations

Professor Siobhan Creanor will lead statistical aspects of the study design and analyses, including supervision of the trial statistician (to be appointed).

13.1 Sample size

Participants: The recruitment target aims to have a sufficient number of participants to provide the operational experience to plan a definitive trial; provide reasonably robust estimates of our feasibility outcomes; and to estimate the variability of the proposed patient-reported outcomes to inform a future sample size calculation. A recruitment target of 60 participants (30 per treatment arm) will allow overall retention rate at 12 weeks to be estimated with precision of ±11%, using an exact 95% confidence interval, based on previously observed retention rates of ~80% for the same

population in the community setting.[42] Assuming a non-differential retention rate of 80% at 12 weeks follow-up, this target will provide follow-up outcome data on ~24 participants per group.

Professionals: Target recruitment is 80% of therapists involved in intervention delivery and invites extended to service managers from each site. This is estimated to be 12 therapists and 4 managers across sites.

13.2 Statistical analysis

Quantitative data

A statistical analysis plan will be finalised ahead of database locking and reporting will follow the CONSORT guidance for pilot and feasibility studies.[57] Primary (descriptive only) analyses will follow the principles of intention to treat.[58]

A CONSORT flow diagram will display data from screening, recruitment and follow-up logs enabling estimation of eligibility, recruitment, consent and follow-up rates.[58] Confidence intervals for recruitment and retention rates will be produced to inform assumptions for planning the definitive trial. Completion rates will be estimated for outcome measures at each time-point, including resource use data. Participants' baseline characteristics will be summarised by allocated group with descriptive statistics (measures of central tendency and dispersion). Patient-reported outcomes, and acceptability will be summarised by allocated group at each follow-up, with descriptive statistics (measures of central tendency and dispersion). Between-group differences, including in changes from baseline, will be reported for the patient-reported outcomes with corresponding confidence intervals.

For patients who do not speak English, the count of pseudo-anonymised patient-reported outcome measures circulated and returned by post will be documented. The content of the patient-reported outcome measure will be described narratively ensuring participant anonymity is preserved (the language versions circulated will not be specified in reporting).

The quantitative analysis will be completed by a trial statistician using well-validated statistical packages after database lock at trial end. A TSDMC will monitor screening and recruitment rates, accruing outcome data and safety reporting. There are no interim analyses planned.

Qualitative data

Qualitative data transcribed verbatim from semi-structured interviews and focus groups will be analysed using a thematic analysis approach.[59] A random sample of intervention audio recordings will be sampled to assess fidelity. High fidelity will be considered as ≥80% of intervention core components (as specified in the intervention manual) fully delivered by the therapist within each session.

Qualitative analysis will be completed by a member of the research team as data becomes available (completion of interviews/focus groups and/or availability of audio recordings to assess fidelity).

13.3 Interim analysis and data monitoring

13.3.1 Discontinuation rules and breaking of randomisation code

The TSDMC will monitor screening and recruitment rates and safety reporting; pooled data will be presented in open reports for review and discussion. Data by allocated group will be provided in separate, closed reports, for review and discussion by the TSDMC. There are no interim analyses planned.

The end of the study is the date when all data queries are resolved, and database locked which is anticipated to be month 17 from funding start. Should the research team/TSDMC deem a need for discontinuation of the trial (e.g., for urgent safety measures), the CI will report the early discontinuation via an NRES End of Study Declaration form to the main REC with a copy to the co-Sponsors.

There are no anticipated circumstances under which the randomisation codes may need to be broken.

13.3.2 Monitoring, quality control and assurance

The trial will be conducted in compliance with the approved protocol, GCP, the UK General Data Protection Regulation and Data Protection Act (2018), the Trust Information Governance Policy (or other local equivalent), the UK Policy Framework for Health and Social Care Research, the Sponsor's Standard Operating Procedures, the Mental Capacity Act 2005, and other applicable regulations as required.

The trial will be led and managed by the CI with the support of the trial manager. This will include co-ordination of all day-to-day aspects of the trial from initial set-up to closeout, including: development of the systems, protocol and other essential documents; REC/HRA submission and amendments; set-up and management of trial management group (TMG) and TSDMC meetings; planning and provision of site initiation visit; day-to-day liaison with site; study monitoring oversight; safety management and reporting, progress reporting to REC, TSDMC, Sponsor and funder; performance of site closure at end of study; provision of end of study notifications to HRA and REC; preparation of files for archiving.

The CI will chair the TMG, which will also include the investigators, PPI representative, trial manager, and trial statisticians meeting at least bi-monthly throughout the trial and more frequently during the earlier phases. The TMG will closely monitor study progress, identifying and addressing practical, scientific, and financial issues as they arise. The TMG will report progress to the Sponsor and the independent TSDMC.

A joint TSDMC will be established prior to trial start to include the CI, trial manager, trial statistician(s), a sponsor representative, as well as the following independent members; Chair, statistician, PPI representative(s), clinician, and health services researcher. The Committee will provide advice, data monitoring (screening and recruitment rates), quality assurance, and safety monitoring (number, nature and outcomes for all SAEs). The Committee may include open and closed sessions. Closed

sessions will not be attended by the chief investigator or trial manager and may be used for data monitoring and/or other discussions at the discretion of the Chair. The Committee will be asked to recommend any necessary actions. It is anticipated that the Committee will meet every six months during the trial period.

A protocol deviation is defined as an unintended departure from the expected conduct of the study protocol, which does not need to be reported to the sponsor. The CI will monitor protocol deviations by monitoring treatment logs completed by treating therapists after each session against the treatment allocation. Any deviations noted will be listed in a deviation log. Significant deviations to the protocol or deviations which are found to frequently recur will be assessed by the CI to see if an amendment to the protocol is required. Any significant deviations to the protocol or recurring deviations will be reported to sponsor and action taken through Corrective and Preventative Actions.

A 'serious breach' is defined as a breach likely to effect to a significant degree:

- The safety or physical or mental integrity of the participants of the trial;
- The scientific value of the trial.

The CI and Sponsor will be notified by the site PI immediately via the study mailbox if a potential serious breach occurs. If the Sponsor determines the breach as serious, the breach will then be reported to the REC Committee with the sponsor in copy within 7 calendar days of the CI being notified.

The trial database will be programmed to generate notifications for possible data entry errors and missing key data points.

Prior to publication, the presentation of results (text, tables and figures) will be reviewed by the trial management group to ensure the anonymity of participants will be preserved. In particular, we will review variable level counts and identify any which are small and in combination with other data could render information potentially identifiable. If this occurs, we will categorize the variable further to ensure anonymity is preserved. If further categorization is not possible, we will suppress the potentially identifiable data and report the extent to which data suppression was employed in the results.

14 Public and patient involvement

We involved patients and carers from proposal conception onwards. In 2020, Sheehan supported the establishment of a PPI group which meets quarterly to discuss Trauma Rehabilitation (Orthopaedic) research for Older People - 'TROOP'. TROOP includes men and women from different ethnic backgrounds who reside at home across England. We were awarded a NIHR Research Design Service Enabling Involvement Fund to support TROOP focus groups and participation in the intervention development workshop for the current project. Members of TROOP informed:

1. Potential outcome measures for a definitive trial (focus group 04/2021). We discussed the EQ-5D-5L, the Nottingham Extended Activities of Daily Living (NEADL) Scale and the University of Alabama Life Space Assessment as prospective primary outcome measures for a future definitive trial. All members were strongly in favour of the EQ-5D-5L as a more

global measure of quality of life. TROOP members suggested the NEADL and University of Alabama Life Space Assessment were valuable additions for understanding how the intervention may work.

2. Participant eligibility (focus group 07/2021). We discussed eligibility criteria for the feasibility trial. All members favoured adopting inclusive criteria which reflect the underlying population. Members proposed revising the lower age limit from 65-years to 60-years. Members agreed with proposed exclusion of those who resided in nursing home/residential care prefracture acknowledging that while these adults should be able to access rehabilitation, the make-up of an appropriate intervention would be different to that proposed here.
3. Intervention (workshops 08 & 09/2021). Members were given the opportunity to provide suggestions for the intervention and feedback on suggestions from others during two workshops. TROOP members suggested starting the intervention 30-days after hip fracture was appropriate and hearing the recovery experience of people who had hip fracture would be beneficial.
4. Review of lay summary and ongoing involvement plans (focus group 10/2021). Members reviewed the plain English summary for the proposal during a meeting and provided feedback over email after the meeting. For ongoing involvement, members suggested front-loading PPI with monthly meetings for first six months and quarterly TROOP meetings thereafter. A TROOP representative will join the trial management committee.

We will continue our active collaboration with PPI members of TROOP for the duration of the project. We will follow the UK Standards for Public Involvement to ensure this collaboration follows best practice and TROOP members continue to be reimbursed appropriately for all contributions.[60]

Philip Bell will take a leadership role for TROOP with respect to the current project and attend bimonthly trial management group meetings.

TROOP members will meet monthly on MS TEAMS for the first six months to ensure they are actively involved in protocol development, ethics, HRA and Local R&D approval processes. In particular, we will seek guidance from TROOP members on the participant information leaflet, consent forms, and proposed approaches for recruitment, data collection, and format of the intervention diary. During this initial six-months, TROOP members will also contribute to plans for the video of older adults who incurred hip fracture. They will support the formulation of interview questions, provide feedback on the proposed set up for the interviews, and provide feedback on the layout of the edited video.

TROOP members will then meet quarterly remotely for 90-minutes. These meetings will include discussion of progress, interpretation of qualitative and quantitative results, and development and dissemination of plain English summaries of the project findings.

TROOP members were offered a practice session to access MS TEAMS prior to attending their first TROOP meeting to ensure they were comfortable with how to join the meeting and that their camera and microphone were working. TROOP members are supported through circulation of materials by email or post -whichever is preferable. We also provided ergonomic pens and visual stress friendly notebooks for TROOP members (as required) to enable them to take notes during TROOP meetings. Philip Bell will be sent materials (including an outline of the format) in advance of

each meeting. He will be offered a pre-meeting with the lead applicant to discuss anything that is not clear.

We have recruited two PPI members independent of TROOP for the TSDMC through NIHR People in Research.

15 Ethical considerations

This trial is funded by the NIHR Research for Patient Benefit Scheme (Sheehan PI), the application for which underwent external peer review. The trial was designed in collaboration with PPI members of TROOP (see Section 14) and TROOP members will have an active role in the management of the trial, as well as in the interpretation and dissemination of the trial findings.

The study requires regulatory approval from the following bodies: NHS REC and HRA. Before any site can enrol patients into the study, the Chief Investigator/Principal Investigator or designee will ensure that the appropriate regulatory approvals have been issued, and NHS Confirmations of Capacity and Capability and Sponsor green lights are in place.

Participants will not be enrolled into the study until informed consent has been obtained, from adults with capacity to consent, by a suitably trained individual (clinicians or trust research staff). Older adults will require capacity to consent due to the safety profile of the intervention (the intervention includes unsupervised outdoor mobility). Non-English language speaking older adults will be supported to consider their enrolment in the trial with the use of local interpreting services.

Participants are free to withdraw from the trial at any point or a participant can be withdrawn by the CI/site PI. See Section 9.9 for further details. No sanctions will follow if the participant decides to leave the research at any time.

To mitigate the risk of ongoing uncertainty at the end of the feasibility trial, we propose progression criteria outlined in Figure 3.[61]

The study will be monitored by the TMG and a TSDMC (See Section 13.3.2). All correspondence with the Sponsor, REC and HRA will be retained.

16 Risk Management

<u>Potential safety risk</u>

Please see Section 11.

<u>Potential distress</u>

Participants may feel they are not making sufficient progress in their recovery. To mitigate this risk, their goal-orientated mobility programme will be tailored to their prefracture abilities. Further, behaviour change techniques including motivation, feedback, social support, as well as professional support (up to 6 visits and 4 telephone calls) have been incorporated into the intervention.

<u>Potential burden</u>

Data collection will pose a potential burden to participants. This collection includes assessments at baseline, 6- and 12- weeks post randomisation, and 6-months (if the timing of randomisation permits 6-month follow-up within the trial data collection window), as well as patient diaries for unsupervised intervention components. We limited proposed data collection from participants to essential data to allow us to assess feasibility and plan for a future definitive trial to minimize potential burden. Assessments will be completed over the telephone/MS TEAMS to reduce the need for travel to complete data collection. Diaries will be discussed with therapist during supervised sessions and not returned to the research team (to minimise need to post materials). Engagement with the intervention will also pose a burden with the need to arrange up to six additional therapist visits at home. To minimise the burden, therapists will arrange all home visits with appropriate notice, and at a time that suits the participants best.

17 Financing

The study is funded by NIHR Research for Patient Benefit [Grant Ref: NIHR204040]. The funder has no competing interests, has had no substantial influence on the planning of the trial, and they will not influence the conduct or reporting of the trial.

18 Insurance

The study is co-sponsored by King's College London (KCL) and NHS Norfolk and Waveney Integrated Care Board. The co-sponsors will, at all times, maintain adequate insurance in relation to the trial. KCL maintain insurance through its own professional indemnity (Clinical Trials) & no-fault compensation, in respect of any claims arising as a result of negligence by its employees, brought by or on behalf of a trial participant. NHS Norfolk and Waveney Integrated Care Board and NHS staff (including honorary contract holders) undertaking research as part of their job role maintain insurance as they are covered by NHS Resolution indemnity schemes if working for a member of those schemes, subject to the usual scheme terms and conditions: https://www.hra.nhs.uk/about-us/news-updates/indemnity-cover-nhs-staff-delivering-research/

19 Publication and Dissemination

19.1 Publication

The study protocol will be made available on ISCRTN.

The results of the study will be published in open-access peer reviewed journals. The findings will be presented at national conferences (British Geriatrics Society; British Orthopaedic Society) and international conferences (Fragility Fracture Network).

We will submit findings to the Hip Fracture NICE Guideline group for subsequent updates (Lamb, Member) and the European Geriatric Medicine Society which has co-authored and an international position statement on hip fracture care processes (President 2018-2020 and Special interest group member, Martin). Internationally, we will deliver a free webinar through the Hip Fracture Recovery

Research Group of the Fragility Fracture Network (Chair Sheehan) to substantiate their position on access to therapy.

We will attract sites for a definitive trial (if feasibility study is successful) by disseminating the feasibility trial findings to UK clinicians via the Chartered Society of Physiotherapists, British Geriatrics Society, Fragility Fracture Network UK.

Following publication of the primary paper, anonymised electronic REDCap data will be exported and stored alongside anonymised transcriptions of interviews indefinitely on the King's Open Research Data System (https://www.kcl.ac.uk/researchsupport/managing/preserve), with proof of ethical approval as a condition of access.

19.2 ***Informing participants***

The results of the study will be summarised in plain English and made available on the team's public and patient involvement group webpage (www.ppitroop.co.uk) and Twitter page (@TROOP_PPI) as well as via the Royal Osteoporosis Society's Bone Matters e-newsletter (circulations in excess of 20,000). Participants will be offered the option of having the plain English summary posted directly to them during the consent process.

20 Disclosure of Interests

The CI has received funding from NIHR Research for Patient Benefit to support this work. The CI also received funding from NIHR Research for Patient Benefit, UK Research & Innovation Future Leaders Fellowship, and the Chartered Society of Physiotherapy Charitable Trust for hip fracture health services research. The CI is the Chair and of the Scientific and Publications Committee of the Falls and Fragility Fracture Audit Programme which managed the National Hip Fracture Database audit at the Royal College of Physicians. This audit captures data related to outdoor mobility status after hip fracture.

21 Figures and Tables

21.1 Figure 1: Schematic overview of study

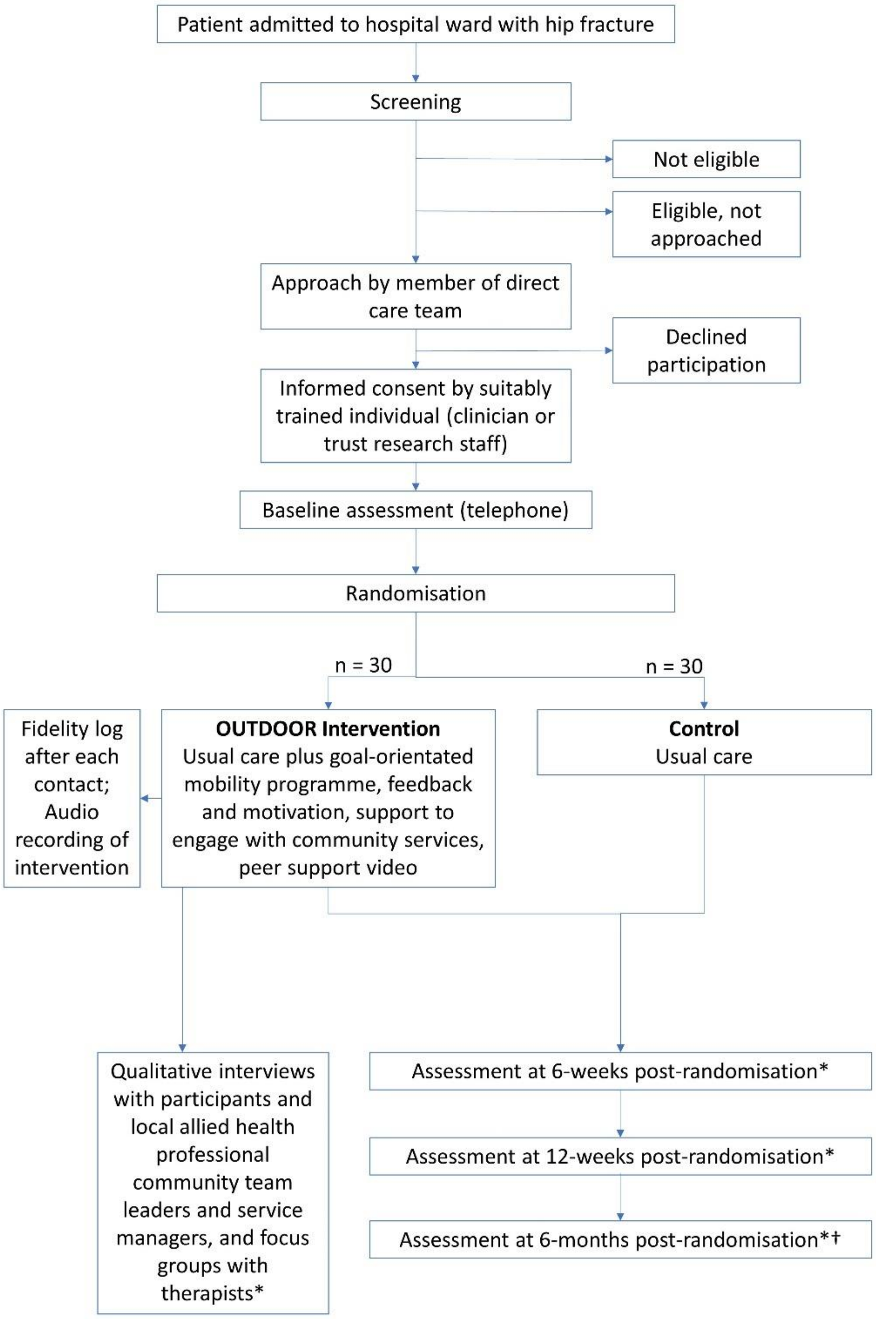

*by telephone or MS TEAMS. †if the timing of randomisation permits this follow-up within the trial data collection window.

21.2 Figure 2: Logic model

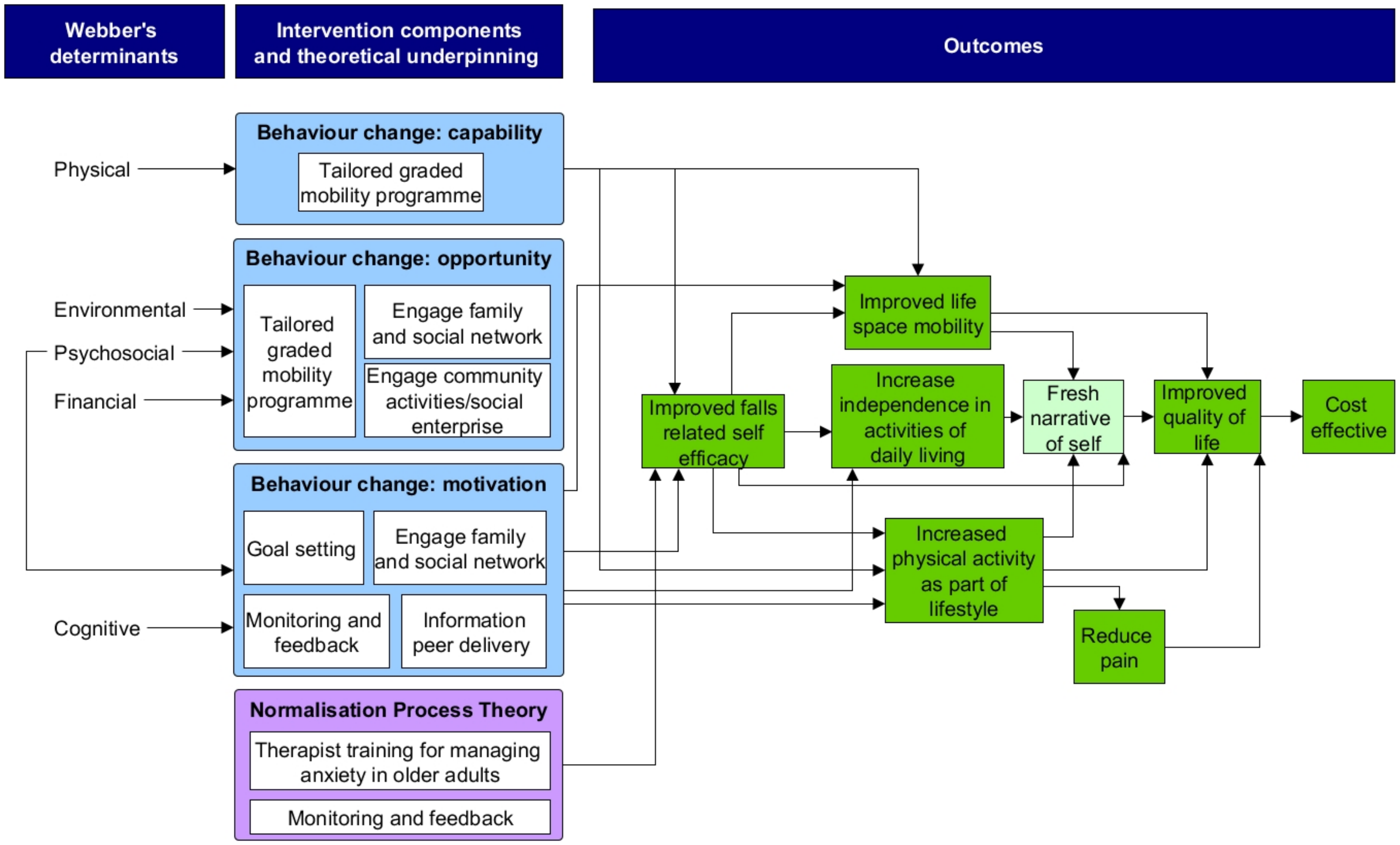

21.3 Figure 3: Progression criteria

	GO	AMEND	STOP
**Recruitment**	≥40% eligible	21-39% eligible	≤20% eligible
**Recruitment**	≥50% eligible recruited	31-49% eligible recruited	≤30% eligible recruited
**Randomisation**	≥70% of those recruited are randomised	49-70% of those recruited are randomised	≤48% of those recruited are randomised
**Acceptability*, participants**	Median of ≥28	Median of 24-27	Median of <24
**Acceptability*, therapists**	Median of ≥28	Median of 24-27	Median of <24
**Fidelity**	≥80% sessions included all intervention components as described	51-79% sessions included all intervention components as described	≤50% sessions included all intervention components as described
**Outcome, 12-weeks**	≥80% completeness of EQ5D at 12-week follow-up	51-79% completeness of EQ5D at 12-week follow-up	≤50% completeness of EQ5D at 12-week follow-up
**Outcome, 6-months†**	≥70% completeness of EQ5D at 6-month follow-up	31-69% completeness of EQ5D at 6-month follow-up	≤30% completeness of EQ5D at 6-month follow-up

*Theoretical Framework of Acceptability questionnaire is comprised of eight questions (affective attitude, burden, perceived effectiveness, intervention coherence, self-efficacy, opportunity costs, general acceptability) each a five-point scale (score range 8-40).

†if the timing of randomisation permits this follow-up within the trial data collection window.

21.4 Table 1: Study time/event matrix

				Time point for collection						
**Event**	**Form**	**Source**	**Completed by**	**Recruitment**	**Baseline**	**Randomisation**	**Intervention (up to 6 visits over 12 weeks)**	**6-weeks**	**12-weeks**	**6-months***
Screening log	Binary and Free text	Patient notes	Site therapist	X						
Approach log	Binary	Patient interview	Site therapist	X						
Contact details	Free text	Patient interview	Site therapist	X						
Consent log	Binary	Patient interview	Site therapist	X						
Age	Numerical	Patient interview	Site therapist		X					
Sex	Binary	Patient interview	Site therapist		X					
Ethnicity	Categorical	Patient notes	Site therapist		X					
Fracture type	Categorical	Patient notes	Site therapist		X					
Surgery type	Categorical	Patient notes	Site therapist		X					
Abbreviated Mental Test	Numerical	Patient notes	Site therapist		X					
Clinical Frailty Scale	Numerical	Patient notes	Site therapist		X					
Residence	Categorical	Patient interview	Research team		X (prefracture)					

Living status	Categorical	Patient interview	Research team		X (prefracture)					
Mobility	Categorical	Patient interview	Research team		X (prefracture)					
Discharge direct to home	Categorical	Patient interview	Research team		X					
EuroQoL EQ-5D-5L	Numerical	Patient questionnaire	Research team		X			X	X	X
Nottingham Extended Activities of Daily Living	Numerical	Patient questionnaire	Research team		X			X	X	X
Falls Efficacy Scale-International	Numerical	Patient questionnaire	Research team		X			X	X	X
Numeric Rating Scale	Numerical	Patient questionnaire	Research team		X			X	X	X
University of Alabama Life Space Assessment	Numerical	Patient questionnaire	Research team		X			X	X	X
Bespoke resource use form	Categorical	Patient questionnaire	Research team		X			X	X	X
Randomisation log	Binary	Computer generated randomisation	Site therapist			X				
Treatment - control	-	-	Site therapist				X			
Treatment - intervention	-	-	Site therapist				X			
Treatment logs	Categorical	Therapist questionnaire	Site therapist				X			
Deviation log	Free text	Therapist questionnaire	Site therapist				X			

Treatment audio recordings	Audio recordings	Audio recordings	Site therapist				X			
Patient diary	Categorical	Patient calendar	Participant				X			
Length of stay	Numerical	Patient notes	Site therapist		X					
Mortality	Binary	Online death records	Research team				X	X	X	X
Readmission	Binary	Patient interview	Research team				X	X	X	X
Readmission diagnosis (as applicable)	Free text	Patient interview	Research team				X	X	X	X
Completion logs	Binary	Therapist questionnaire	Site therapist						X	
Patient semi-structured interviews	Free text	Patient interview	Research team						X	
Patient acceptability	Categorical	Questionnaire	Research team						X	
Family/friends engaged	Binary	Patient interview	Research team						X	
Therapist semi-structured interviews	Free text	Therapist interview	Research team						X	
Therapist acceptability	Categorical	Questionnaire	Research team						X	

* if the timing of randomisation permits this follow-up within the trial data collection window; orange fill indicates events applicable only to the intervention arm.

22 Appendix 1 Information with regards to Safety Reporting in Non-CTIMP Research

	Who	When	How	To Whom
**SAE**	Chief Investigator	Report to Sponsor within 24 hours of learning of the event<br><br>Report to the MREC within 15 days of learning of the event	SAE Report form for Non-CTIMPs, available from NRES website.	Sponsor and MREC
**Urgent Safety Measures**	Chief Investigator	Contact the Sponsor Immediately<br><br>MREC to be notified Within 3 days	By phone/email<br><br>Initial notification must set out the reasons for the urgent safety measures and the plan for further action.<br><br>Where required, Substantial amendment should be submitted as soon as it is possible to do so.	Main REC and Sponsor<br><br>MREC will aim to give a formal opinion on the substantial amendment within 28 calendar days but will give an opinion in no more than 35 days.
**Minor Protocol deviations or GCP non-compliance**	Chief Investigator	Contact the Sponsor as soon as possible after learning of the event	By email using the file note template, protocol deviation log and/or file note log templates	Sponsor<br><br>Voluntary notification to REC manager and to breaches@hra.nhs.uk for information
**Serious Breaches**	Chief Investigator	Contact the Sponsor immediately<br><br>MREC to be notified within 7 days of Sponsor notification	By email including details of when the breach occurred, the location, who was involved, the outcome and any information given to participants. An explanation should be given, and the REC informed what further action the sponsor plans to take.	Main REC and Sponsor<br><br>Reports provided may be referred to the Health Research Authority breaches@hra.nhs.uk for consideration by the Main REC
**Progress Reports**	Chief Investigator	Annually (starting 12 months after the date of favourable opinion)	Annual Progress Report Form (non-CTIMPs) available from the NRES website	Main REC

**<u>Declaration of the conclusion or early termination of the study</u>**	Chief Investigator	Within 90 days (conclusion)<br><br>Within 15 days (early termination)<br><br>*The end of study should be defined in the protocol*	End of Study Declaration form available from the NRES website	Main REC with a copy to be sent to the sponsor
**<u>Summary of final Report</u>**	Chief Investigator	Within one year of conclusion of the Research	No Standard Format However, the following Information should be included:-<br>Where the study has met its objectives, the main findings and arrangements for publication or dissemination including feedback to participants	Main REC with a copy to be sent to the sponsor

23 References

1. Royal College of Physicians. National Hip Fracture Database (NHFD) Annual Report 2019, 2019. Available at: https://www.nhfd.co.uk/20/hipfracturer.nsf/docs/2019Report [Accessed on 17/03/2022]
2. Borges FK et al. Accelerated surgery versus standard care in hip fracture (HIP ATTACK): an international, randomised, controlled trial. The Lancet. 2020 Feb 29;395(10225):698-708.
3. Haentjens P, Magaziner J, Colon-Emeric CS, et al. Meta-analysis: excess mortality after hip fracture among older women and men. *AnnInternMed* 2010;152(6):380-90.
4. Nanjayan SK, John J, Swamy G, et al. Predictors of change in 'discharge destination' following treatment for fracture neck of femur. *Injury* 2014;45(7):1080-4. doi: 10.1016/j.injury.2014.02.005 [published Online First: 2014/03/13]
5. Dreinhofer KE, Mitchell PJ, Begue T, et al. A global call to action to improve the care of people with fragility fractures. *Injury* 2018;49(8):1393-97. doi: 10.1016/j.injury.2018.06.032 [published Online First: 2018/07/10]
6. Beer N, et al. Patient perspectives of recovery after hip fracture: a systematic review and qualitative synthesis. Disability and Rehabilitation. 2021 Sep 16:1-6.
7. World Health Organisation. World Report on Ageing and Health. In: WHO, ed. Geneva, Switzerland, 2016. Available at: https://apps.who.int/iris/handle/10665/186463 [Accessed on 17/03/2022]
8. Griffiths F, Mason V, Boardman F, et al. Evaluating recovery following hip fracture: a qualitative interview study of what is important to patients. *BMJ Open* 2015;5(1):e005406. doi: 10.1136/bmjopen-2014-005406
9. Zidén L, Scherman MH, Wenestam C-g. The break remains–elderly people's experiences of a hip fracture 1 year after discharge. *Disability and rehabilitation* 2010;32(2):103-13.
10. Goubar A, et al. The 30-day survival and recovery after hip fracture by timing of mobilization and dementia: a UK database study. The Bone & Joint Journal. 2021 Jul 1;103(7):1317-24.
11. Almilaji et al., Physiotherapy Provision and Outcomes of Acute Hospital Stay: Secondary Analysis of the UK Physiotherapy Hip Fracture Sprint Audit 2022. Physiotherapy 2023.
12. National Institute for Health and Care Excellence. The management of hip fracture in adults. London: Royal College of Physicians (UK), 2011. Available at: https://www.nice.org.uk/guidance/cg124 [Accessed on 17/03/2022]
13. Royal College of Physicians. Recovering after a hip fracture: helping people understand physiotherapy in the NHS. Physiotherapy 'Hip Sprint' audit report In: RCP, ed. London, 2017. Available at: https://www.rcplondon.ac.uk/projects/outputs/recovering-after-hip-fracture-helping-people-understand-physiotherapy-nhs [Accessed on 17/03/2022]
14. McDonough CM, Harris-Hayes M, Kristensen MT, et al. Physical Therapy Management of Older Adults With Hip Fracture. *J Orthop Sports Phys Ther* 2021;51(2):CPG1-CPG81. doi: 10.2519/jospt.2021.0301 [published Online First: 2021/02/02]
15. Williams N H, Roberts J L, Din N U, et al. Chapter 4 Survey of hip fracture centres, physiotherapists and occupational therapists in Developing a multidisciplinary rehabilitation package following hip fracture and testing in a randomised feasibility study: Fracture in the Elderly Multidisciplinary Rehabilitation (FEMuR). In: Assessment HT, ed., 2017.
16. Portegijs E, Keskinen KE, Eronen J, et al. Older Adults' Physical Activity and the Relevance of Distances to Neighborhood Destinations and Barriers to Outdoor Mobility. *Front Public Health* 2020;8:335. doi: 10.3389/fpubh.2020.00335 [published Online First: 2020/08/28]
17. Jacobs JM, Cohen A, Hammerman-Rozenberg R, et al. Going outdoors daily predicts long-term functional and health benefits among ambulatory older people. *J Aging Health* 2008;20(3):259-72. doi: 10.1177/0898264308315427 [published Online First: 2008/03/12]

18. Smith TO, Dainty JR, MacGregor A. Trajectory of social isolation following hip fracture: an analysis of the English Longitudinal Study of Ageing (ELSA) cohort. *Age Ageing* 2018;47(1):107-12. doi: 10.1093/ageing/afx129 [published Online First: 2017/10/07]
19. Saletti-Cuesta L, Tutton E, Langstaff D, et al. Understanding informal carers' experiences of caring for older people with a hip fracture: a systematic review of qualitative studies. *Disabil Rehabil* 2018;40(7):740-50. doi: 10.1080/09638288.2016.1262467 [published Online First: 2016/12/16]
20. Osnes EK, Lofthus CM, Meyer HE, et al. Consequences of hip fracture on activities of daily life and residential needs. *Osteoporos Int* 2004;15(7):567-74. doi: 10.1007/s00198-003-1583-0 [published Online First: 2004/01/20]
21. Hajek A, Brettschneider C, Lange C, et al. Longitudinal Predictors of Institutionalization in Old Age. *PLoS One* 2015;10(12):e0144203. doi: 10.1371/journal.pone.0144203 [published Online First: 2015/12/15]
22. Leal J, Gray AM, Prieto-Alhambra D, et al. Impact of hip fracture on hospital care costs: a population-based study. *Osteoporos Int* 2016;27(2):549-58. doi: 10.1007/s00198-015-3277-9 [published Online First: 2015/08/20]
23. Ahmed T, Curcio CL, Auais M, et al. Falls and life-space mobility: longitudinal analysis from The International Mobility in Aging Study. *Aging Clin Exp Res* 2021;33(2):303-10. doi: 10.1007/s40520-020-01540-0 [published Online First: 2020/04/10]
24. Webber SC, Porter MM, Menec VH. Mobility in older adults: a comprehensive framework. *Gerontologist* 2010;50(4):443-50. doi: 10.1093/geront/gnq013 [published Online First: 2010/02/11]
25. Michie S, Richardson M, Johnston M, et al. The behavior change technique taxonomy (v1) of 93 hierarchically clustered techniques: building an international consensus for the reporting of behavior change interventions. *Ann Behav Med* 2013;46(1):81-95. doi: 10.1007/s12160-013-9486-6 [published Online First: 2013/03/21]
26. Murray E, Treweek S, Pope C, et al. Normalisation process theory: a framework for developing, evaluating and implementing complex interventions. *BMC Med* 2010;8:63. doi: 10.1186/1741-7015-8-63 [published Online First: 2010/10/22]
27. Sheehan KJ, Fitzgerald L, Lambe K, Martin FC, Lamb SE, Sackley C. Effectiveness of community-based rehabilitation interventions incorporating outdoor mobility on ambulatory ability and falls-related self-efficacy after hip fracture: a systematic review and meta-analysis. Archives of osteoporosis. 2021 Dec;16(1):1-6.
28. James Lind Alliance. Broken Bones in Older people - Musculoskeletal Injury: fragility fracture of the lower limb and pelvis Top 10, 2018. Available at: https://www.jla.nihr.ac.uk/priority-setting-partnerships/broken-bones-in-older-people/ [Accessed on 17/03/2022]
29. Geohagen O, Hamer L, Lowton A, Guerra S, Milton-Cole R, Ellery P, Martin FC, Lamb SE, Sackley C, Sheehan KJ. The effectiveness of rehabilitation interventions including outdoor mobility on older adults' physical activity, endurance, outdoor mobility and falls-related self-efficacy: systematic review and meta-analysis. Age and ageing. 2022 Jun;51(6):afac120.
30. Mangione KK, Craik RL, Tomlinson SS, et al. Can elderly patients who have had a hip fracture perform moderate- to high-intensity exercise at home? *Phys Ther* 2005;85(8):727-39. [published Online First: 2005/07/29]
31. Crotty M, Whitehead CH, Gray S, et al. Early discharge and home rehabilitation after hip fracture achieves functional improvements: a randomized controlled trial. *Clin Rehabil* 2002;16(4):406-13. doi: 10.1191/0269215502cr518oa [published Online First: 2002/06/14]
32. Orwig DL, Hochberg M, Yu-Yahiro J, et al. Delivery and outcomes of a yearlong home exercise program after hip fracture: a randomized controlled trial. *Arch Intern Med* 2011;171(4):323-31. doi: 10.1001/archinternmed.2011.15 [published Online First: 2011/03/02]

33. Ziden L, Kreuter M, Frandin K. Long-term effects of home rehabilitation after hip fracture - 1-year follow-up of functioning, balance confidence, and health-related quality of life in elderly people. *Disabil Rehabil* 2010;32(1):18-32. doi: 10.3109/09638280902980910 [published Online First: 2009/11/21]
34. Pol MC, Ter Riet G, van Hartingsveldt M, et al. Effectiveness of sensor monitoring in a rehabilitation programme for older patients after hip fracture: a three-arm stepped wedge randomised trial. *Age Ageing* 2019;48(5):650-57. doi: 10.1093/ageing/afz074 [published Online First: 2019/06/18]
35. Ziden L, Frandin K, Kreuter M. Home rehabilitation after hip fracture. A randomized controlled study on balance confidence, physical function and everyday activities. *Clin Rehabil* 2008;22(12):1019-33. doi: 10.1177/0269215508096183 [published Online First: 2008/12/05]
36. Varas AB, Cordoba S, Rodriguez-Andonaegui I, et al. Effectiveness of a community-based exercise training programme to increase physical activity level in patients with chronic obstructive pulmonary disease: A randomized controlled trial. *Physiother Res Int* 2018;23(4):e1740. doi: 10.1002/pri.1740 [published Online First: 2018/09/01]
37. de Roos P, Lucas C, Strijbos JH, et al. Effectiveness of a combined exercise training and home-based walking programme on physical activity compared with standard medical care in moderate COPD: a randomised controlled trial. *Physiotherapy* 2018;104(1):116-21. doi: 10.1016/j.physio.2016.08.005 [published Online First: 2017/08/15]
38. Clemson L, Cumming RG, Kendig H, et al. The effectiveness of a community-based program for reducing the incidence of falls in the elderly: a randomized trial. *J Am Geriatr Soc* 2004;52(9):1487-94. doi: 10.1111/j.1532-5415.2004.52411.x [published Online First: 2004/09/03]
39. Logan PA, Armstrong S, Avery TJ, et al. Rehabilitation aimed at improving outdoor mobility for people after stroke: a multicentre randomised controlled study (the Getting out of the House Study). *Health Technol Assess* 2014;18(29):vii-viii, 1-113. doi: 10.3310/hta18290 [published Online First: 2014/05/09]
40. Logan PA, Gladman JR, Avery A, et al. Randomised controlled trial of an occupational therapy intervention to increase outdoor mobility after stroke. *BMJ* 2004;329(7479):1372-5. doi: 10.1136/bmj.38264.679560.8F [published Online First: 2004/11/27]
41. Daniela Kahlert NE. Out-of-Home Mobility and Social Participation of Older People: a Photo-Based Ambulatory Assessment Study. *Journal of Population Ageing* 2020;13:547-60.
42. Williams NH, Roberts JL, Din NU, et al. Fracture in the Elderly Multidisciplinary Rehabilitation (FEMuR): a phase II randomised feasibility study of a multidisciplinary rehabilitation package following hip fracture. *BMJ Open* 2016;6(10):e012422. doi: 10.1136/bmjopen-2016-012422 [published Online First: 2016/10/07]
43. Rantakokko M, Manty M, Iwarsson S, et al. Fear of moving outdoors and development of outdoor walking difficulty in older people. *J Am Geriatr Soc* 2009;57(4):634-40. doi: 10.1111/j.1532-5415.2009.02180.x [published Online First: 2009/04/28]
44. Merom D, Gebel K, Fahey P, et al. Neighborhood walkability, fear and risk of falling and response to walking promotion: The Easy Steps to Health 12-month randomized controlled trial. *Prev Med Rep* 2015;2:704-10. doi: 10.1016/j.pmedr.2015.08.011 [published Online First: 2016/02/05]
45. Delbecq AL, Vandeven AH. Group Process Model for Problem Identification and Program Planning. *Journal of Applied Behavioral Science* 1971;7(4):466-&. doi: Doi 10.1177/002188637100700404
46. Miller WR, Rose GS. Motivational interviewing and decisional balance: contrasting responses to client ambivalence. *Behav Cogn Psychother* 2015;43(2):129-41. doi: 10.1017/S1352465813000878 [published Online First: 20131111]

47. Haywood KL, Griffin XL, Achten J, et al. Developing a core outcome set for hip fracture trials. *Bone Joint J* 2014;96-B(8):1016-23. doi: 10.1302/0301-620X.96B8.33766
48. Janssen MF, Pickard AS, Golicki D, et al. Measurement properties of the EQ-5D-5L compared to the EQ-5D-3L across eight patient groups: a multi-country study. *Qual Life Res* 2013;22(7):1717-27. doi: 10.1007/s11136-012-0322-4 [published Online First: 2012/11/28]
49. Baker PS, Bodner EV, Allman RM. Measuring life-space mobility in community-dwelling older adults. *J Am Geriatr Soc* 2003;51(11):1610-4. doi: 10.1046/j.1532-5415.2003.51512.x [published Online First: 2003/12/23]
50. Harwood RH, Ebrahim S. The validity, reliability and responsiveness of the Nottingham Extended Activities of Daily Living scale in patients undergoing total hip replacement. *Disabil Rehabil* 2002;24(7):371-7. doi: 10.1080/10.1080/09638280110101541 [published Online First: 2002/05/23]
51. Yardley L, Beyer N, Hauer K, et al. Development and initial validation of the Falls Efficacy Scale-International (FES-I). *Age Ageing* 2005;34(6):614-9. doi: 10.1093/ageing/afi196 [published Online First: 2005/11/04]
52. Haefeli M, Elfering A. Pain assessment. *Eur Spine J* 2006;15 Suppl 1(Suppl 1):S17-24. doi: 10.1007/s00586-005-1044-x [published Online First: 20051201]
53. Sekhon M, Cartwright M, Francis JJ. Acceptability of healthcare interventions: an overview of reviews and development of a theoretical framework. *BMC Health Serv Res* 2017;17(1):88. doi: 10.1186/s12913-017-2031-8 [published Online First: 2017/01/28]
54. Saunders B, Sim J, Kingstone T, et al. Saturation in qualitative research: exploring its conceptualization and operationalization. *Qual Quant* 2018;52(4):1893-907. doi: 10.1007/s11135-017-0574-8 [published Online First: 2018/06/26]
55. Rockwood K, Song X, MacKnight C, et al. A global clinical measure of fitness and frailty in elderly people. *CMAJ* 2005;173(5):489-95. doi: 10.1503/cmaj.050051 [published Online First: 2005/09/01]
56. Borrelli B. The Assessment, Monitoring, and Enhancement of Treatment Fidelity In Public Health Clinical Trials. *J Public Health Dent* 2011;71(s1):S52-S63. doi: 10.1111/j.1752-7325.2011.00233.x [published Online First: 2011/04/19]
57. Eldridge SM, Chan CL, Campbell MJ, et al. CONSORT 2010 statement: extension to randomised pilot and feasibility trials. *Pilot Feasibility Stud* 2016;2:64. doi: 10.1186/s40814-016-0105-8 [published Online First: 2016/12/15]
58. Detry MA, Lewis RJ. The intention-to-treat principle: how to assess the true effect of choosing a medical treatment. *JAMA* 2014;312(1):85-6. doi: 10.1001/jama.2014.7523
59. Braun V, Clarke V. Using thematic analysis in psychology. *Qualitative research in psychology* 2006;3(2):77-101.
60. UK Standards for Public Involvement in Research 2016 [Available from: https://sites.google.com/nihr.ac.uk/pi-standards/home.
61. Avery KN, Williamson PR, Gamble C, et al. Informing efficient randomised controlled trials: exploration of challenges in developing progression criteria for internal pilot studies. *BMJ Open* 2017;7(2):e013537. doi: 10.1136/bmjopen-2016-013537 [published Online First: 2017/02/19]
