# Supplementary material for: Protocol for a feasibility randomised controlled trial of the ‘Outdoor’ mobility intervention for older adults after hip fracture
Source: PLoS One. 2024 Aug 12;19(8):e0306871. doi: 10.1371/journal.pone.0306871 (PMC12139654; doi:10.1371/journal.pone.0306871)
Supplement: S3 File — (PDF) [file pone.0306871.s003.pdf]

CONSENT FORM FOR PARTICIPANTS IN RESEARCH PROJECTS

Please complete this form after you have read the Information Sheet and/or listened to an explanation about the research

**Title of project**: Outdoor Mobility After Hip Fracture: A Feasibility Randomised Controlled Trial	
**Name of Chief Investigator:** Emma Godfrey	
**Name of Principal Investigator at local site:**	**Initial**
1. I confirm that I have read and understood the information sheet version 2 dated 11/10/2023 for the above project. I have had the opportunity to consider the information and ask questions which have been answered to my satisfaction.	
2. I consent voluntarily to be a participant in this project and understand that I can refuse to take part and can withdraw from the project at any time, without having to give a reason, without my medical care or legal rights being affected. I agree that if I decide to withdraw, data collected up until the point of withdrawal will be retained by the research team.	
3. I understand my personal information will be processed for the purposes explained to me in the Information Sheet. I understand that such information will be handled under the terms of UK data protection law, including the UK General Data Protection Regulation (UK GDPR) and the Data Protection Act 2018.	
4. I understand that relevant sections of my medical notes and data collected during the study, may be looked at by individuals from King's College London, from regulatory authorities or from the NHS Trust where it is relevant to my taking part in this research or for monitoring and audit purposes. I give permission for these individuals to have access to my records.	
5. I understand that confidentiality and anonymity will be maintained, and it will not be possible to identify me in any research outputs.	
6. I understand that anonymous data that does not identify me may be archived and shared publicly for research purposes.	
7. I consent to audio recording of sessions with therapists.	
8. I consent to audio recording of sessions with therapists to be shared with third parties for transcription purposes.	
9. I agree to be contacted by telephone to schedule an interview about the study.	
10. I agree for this interview to be audio recorded and transcribed by an external company.	
11. I agree for my direct quotes, that do not identify me, to be used in the study reporting.	
12. I consent to my data being shared with the University of Exeter as outlined in the participant information sheet. The University of Exeter will have signed an agreement with Kings College London.	
13. I agree that the research team may use my data for future research and understand that any such use of identifiable data would be reviewed and approved by a research ethics committee as required. (In such cases, as with this project, data would not be identifiable in any report).	

The following clauses are OPTIONAL. Initial Yes (Y) or No (N)	Y	N
14. I wish to receive a copy of the final report. I agree to the researchers using my contact details from this purpose.		
15. I agree that the researcher may retain my contact details for 5 years so that I may be contacted in the future by King's College London researchers who would like to invite me to participate in future studies of a similar nature.		

______________________ ______________________ ______________________

Name of Participant **Date** **Signature**

______________________ ______________________ ______________________

Name of Researcher **Date** **Signature**

When completed: 1 for participant; 1 for researcher site file; 1 to be kept in medical notes
