# Supplementary material for: Protocol for a feasibility randomised controlled trial of the ‘Outdoor’ mobility intervention for older adults after hip fracture
Source: PLoS One. 2024 Aug 12;19(8):e0306871. doi: 10.1371/journal.pone.0306871 (PMC12139654; doi:10.1371/journal.pone.0306871)
Supplement: S4 File — (PDF) [file pone.0306871.s004.pdf]

Participants' experiences of the OUTDOOR intervention

Topic guide

Thank you for agreeing to take part in this interview today, which focus on your experiences of taking part in the OUTDOOR study.

The OUTDOOR intervention is additional rehabilitation at home designed for people who have surgery to repair a broken hip. The aim of the study was to evaluate whether it is possible to deliver the OUTDOOR intervention in the NHS. If so, we will move forward to a bigger study to see whether the approach is better than what is currently offered within the NHS.

This interview will last no more than 45 minutes. I will be taking notes during the interview, and I would also like to record the interview so that I do not miss anything you say if that is OK.

Everything that you tell me will be kept completely confidential unless it is felt there was a risk of harm to yourself or others, in which case standard safeguarding procedures will be followed. When we write up the results you will not be identified in any way.

SECTION A – FOR ALL

1. I'd like to start off by asking you about your general impression (experiences) of the OUTDOOR study?
    - *Tell me about what you received*
    - *Interviewer to clarify what was the OUTDOOR study and what was usual care*
2. What motivated or encouraged you to take part in the OUTDOOR study?
3. Can you talk me through how you got into the study?
    - How did it feel that you were put in the group that got extra rehabilitation?
    - Would you have felt differently if you were put in the group that did not get the extra rehabilitation?
    - Could we have done anything differently?
4. What were you expecting from the OUTDOOR study?
    - *To what extent would you say your expectations were met?*
    - *What do you think made it easy to take part in the OUTDOOR study?*

- *What do you think made it difficult to take part?*

5. Did you talk to any other patients or carers about the OUTDOOR intervention?
6. As part of the study, we had to ask you a lot of questions at the beginning, after 6-weeks, and then again 12-weeks later *[and 6-months later if included in 6-month follow-up].* How did you find answering these questions?
    - *What did you think about the number of questions asked?*
    - *How did answering the questions fit in with your routine?*
    - *Any other comments about answering the questions?*

I would like to now talk to you about the OUTDOOR study in more detail.

7. What aspects of the OUTDOOR study did you find particularly helpful?

- *E.g., what did you think about the content*
- *having the session delivered by the therapist/therapy assistant?*
- *did you find anything in the sessions unhelpful?*

---

1. What did you think of the video where people shared their experience of recovery after hip fracture?
    - *How useful?*
    - *What did you think about how you accessed it?*
2. What did you think of the goals that you set for your outdoor mobility?
    - *To what extent did they meet your expectations for this phase of your recovery?*
3. How did you feel about the extra sessions you had with the therapist?
    - *How convenient were the for you?*
    - *To what extent did they meet your expectations for your outdoor mobility recovery?*
4. How did you feel about practicing outdoor mobility yourself?
5. Did you ask friends and family to support you to practice your outdoor mobility?
    - *How did you feel about asking them?*
    - *What did their support look like?*
    - *Do you think this was an important for your outdoor mobility recovery?*
6. What are your thoughts and experiences of completing the outdoor mobility by yourself?

- *To what extent was it easy/difficult to remember and complete?*
- *To what extent was finding time to practice your outdoor mobility an issue?*
- *To what extent did the environment impact your ability to practice your outdoor mobility? For example, if you have stairs to get in and out, or the weather.*
- *To what extent did you adapt any of your outdoor mobility?*

7. How did you motivate yourself to practice by yourself?

8. How did you feel about discussions with the therapist about how you felt during and after practicing your outdoor mobility by yourself?
    - *Did you use the diary provided to take note of when you practiced? If so, how helpful was this?*
    - *To what extent did the therapist discussion influence your ability to practice by yourself?*

9. Did you receive telephone calls from the therapist during the study to see how you were getting on with your practice?
    - *If yes, to what extend did the telephone calls influence your outdoor mobility practice?*

10. To what extent did the therapist help you to identify community activities that you could join after the study ended?
    - *Have you joined any groups as a result?*
        - *i. If so, which ones? To what extent did the therapist discussion influence your decision to join the group?*
        - *ii. If no, why not?*

11. Would you have liked more sessions with the therapist?

12. How could we improve the rehabilitation to better support people to get back to going outside of their home after surgery to fix a broken hip?

13. To what extent did the OUTDOOR study give you any new tips about how to manage your recovery?
    - *How have you incorporated any of it into your everyday life?*

14. Overall, how acceptable did you find the treatment you have received?

15. Is there anything else you would like to add?

We are now at the end of the interview.

Thank you for taking part.

Therapist experiences and acceptability of receiving the OUTDOOR training and delivering the OUTDOOR rehabilitation intervention

Topic guide

Thank you for taking the time to participate in this online focus group. The focus group will last an hour. Before starting, I'll give you a summary of the structure. First we will focus on your experience and thoughts of the OUTDOOR ***training*** you received. Second we will discuss your experience of ***delivering*** the OUTDOOR rehabilitation, and third, we will ask your thoughts about the ***trial processes*** involved with delivering the OUTDOOR intervention.

We will be take notes during the focus group, and would also like to record it so that we do not miss anything you say if that is OK. In respect for each other, we ask that only one individual speak at a time in the group. We remind you that responses made by all participants must be kept confidential.

Part 1: Acceptability of OUTDOOR training

We will first focus on the training you received on the OUTDOOR study, and then the training on concerns about falls.

1. Can you talk me through your overall experience of the OUTDOOR intervention training and support materials you received?
   - *What are your thoughts on the training session?*
   - *what are your thoughts on the clinician manual that was provided?*
   - *What are your thoughts on the patient support materials? (i.e. patient diary)*

2. How acceptable did you find the OUTDOOR training?
   - *We are interested in what acceptability might mean to you, so any initials thoughts you might have?*

3. Can you describe to me what you feel was the purpose of you attending the training?
   - *How do you think attending the training sessions may help you deliver the OUTDOOR intervention?*

4. Was there anything that you particularly liked or disliked about the training?
   - *Are there any parts that stand out?*

5. To what extent do you think attending the training prepared you to deliver the OUTDOOR intervention?
   - *Can you tell me more?*

6. Did attending the training sessions interfere with your other workload priorities?
   - *If so, can you tell me more?*

7. Having completed the training, how would you say the intervention fits with your general approach to patient care?
   - *can you tell me more?*
   - *Do you feel that there are any ethical issues that need to be considered?*

8. Can you think of any ways that the training you received could be changed?

Moving on to your training on motivational interviewing and behaviour change techniques.

1. Prior to the OUTDOOR intervention, have you had any training in these techniques?
   - *Can you tell me more?*

2. Following on from that, have you had any experience of changing patients behaviour?
   a. *If so can you tell me more?*

3. Was there anything that you particularly liked or disliked about the training?
   a. *Are there any parts that stand out?*

4. In your opinion how easy or difficult was it to incorporate this training into the OUTDOOR intervention?

   - *What was easy/difficult?*
   - *How much effort was required to include addressing concerns about falls in the OUTDOOR rehabilitation intervention?*
   - *Can you tell me more?*

5. To what extent did you engage with the additional post-training resources?
   a. *How helpful were the 'How-to' videos?*

6. How would you say the training fits with your approach to patient care?
   - *can you tell me more?*

7. Can you think of any ways that the training or additional materials you received could be changed?

Part 2: Acceptability of delivering the OUTDOOR rehabilitation intervention

So now we will move onto part two of the focus group. Some of these questions are very similar but we are interested in your thoughts on your experience and acceptability of delivering the OUTDOOR intervention.

9. Can you talk me through your overall experience of delivering the OUTDOOR rehabilitation intervention?

10. To what extent did you find delivering the OUTDOOR rehabilitation intervention acceptable?

11. Can you briefly tell me how you think delivering the OUTDOOR rehabilitation intervention will help patients with their recovery?
    a. *Can you tell me more?*

12. Was there anything in particularly that you liked or disliked about delivering the OUTDOOR intervention?
    b. *Are there any parts that stand out?*
    c. *Follow on with prompts if participant mentions specifics e.g. showing the video*

13. In your opinion how easy or difficult was it to deliver the OUTDOOR intervention?
    d. *What was easy/difficult?*
    e. *How much effort was required to deliver the OUTDOOR rehabilitation intervention?*
    f. *Can you tell me more?*

14. To what extent do you feel delivering the OUTDOOR rehabilitation intervention may or may not have any ethical implications for patient care?
    g. *Can you tell me more?*
    h. *How challenging was it to only focus on delivering the OUTDOOR rehabilitation when seeing the participant?*

15. Did delivering the OUTDOOR rehabilitation intervention interfere with your other work load priorities?
    i. *If so, can you give me an example?*

16. How confident did you feel about delivering the OUTDOOR rehabilitation intervention?
    j. *Can you tell me more?*

17. Did you adapt any parts of the OUTDOOR rehabilitation intervention?
    k. *If yes, can you give me an example (why and what)*

18. What type of on-going training do you think will help with the delivery of the OUTDOOR intervention?
    - *Can you tell me more?*

19. In your opinion, what influenced patients adhering to the unsupervised components of the intervention?

- *Can you give me an example?*
- *How do you think patient adherence could be improved?*

20. Do you think you used the OUTDOOR intervention or components of the intervention on participants in the usual care group? or patients not enrolled in the trial?
   - *If so, can you give me an example?*
   - *If so, how do you think this could be avoided in a future trial?*

Part 3. Trial processes

We're coming onto the final section of the focus group. Here we are interested in hearing about your thoughts on the trial processes associated with the OUTDOOR intervention, specifically if there are any ways in which the processes could be improved.

1. Could you briefly describe the process of by which you were notified of a patients enrolment?
   I. Do you think this could be improved? If so, how?

2. Could you briefly describe the trial processes for your site once the patient had been randomised to intervention or control arm?
   - *How was the patients consent and allocation communicated to you?*
   - *Do you think this could be improved?*

3. How easy or difficult did you find it to complete/ fill in the paperwork associated with the trial?
   - *Can you give me an example?*
   - *Do you think this could be improved?*

4. With regards to the intervention data collection logs that were provided, how useful were they as a prompt?
   - *Can you tell more?*
   - *Do you think there was anything missing from the intervention data collection logs?*

5. How easy or difficult did you find it to record the intervention sessions and upload the files to the server?
   a. Can you tell more?

6. What was the process or reporting adverse events?

7. Do you have any additional comments regarding the OUTDOOR intervention?

Thank – you that brings us to the end of the focus group.

Managers experience and acceptability of the OUTDOOR rehabilitation intervention

Topic guide

Thank you for taking the time to participate in this interview. The interview will last between 30 and 45 minutes. Before starting, I'll give you a summary of the structure. First we will focus on your experience and thoughts on the OUTDOOR intervention itself. Second we will discuss your experience and thoughts on the trial processes involved with delivering the OUTDOOR intervention.

I will be take notes during the interview, and would also like to record it so that I do not miss anything you say if that is OK. Everything that you tell me will be kept completely confidential unless it is felt there was a risk of harm to yourself or others, in which case standard safeguarding procedures will be followed. When we write up the results you will not be identified in any way.

Part 1: Acceptability of the OUTDOOR rehabilitation intervention

21. To what extent did you find supporting the delivery of the OUTDOOR rehabilitation intervention acceptable?

22. In your opinion how easy or difficult was it to support the delivery of the OUTDOOR intervention?
    - m. *What was easy/difficult?*
    - n. *How much effort was required to deliver the OUTDOOR rehabilitation intervention?*
    - o. *Time, staffing, costs*
    - p. *Can you tell me more?*

23. Was there anything in particularly that you liked or disliked about supporting the delivery of the OUTDOOR intervention?
    - q. *Follow on with prompts if participant mentions specifics*

24. Do you think the OUTDOOR rehabilitation intervention will help patients with their recovery over and above what is currently offered?
    - r. *Can you tell me more?*

25. To what extent do you feel delivering the OUTDOOR rehabilitation intervention may or may not have any ethical implications for patient care?
    - s. *Can you tell me more?*

26. Did supporting the delivery of the OUTDOOR rehabilitation intervention interfere with your other work load priorities?
    - t. *If so, can you give me an example?*

27. Do you think on-going training of your therapy team in psychological strategies to support the delivery of the OUTDOOR intervention is acceptable?
    - *Can you tell me more?*

28. If the OUTDOOR intervention is found to be effective at improving patient outcomes in a future study, do you think you would look to incorporate it into usual practice?
    - *If no, why?*
    - *If yes, what barriers/facilitators do you envisage?*
    - *Time, staffing, costs, other caseload priorities*

Part 2. Trial processes

Here we are interested in hearing about your thoughts on the trial processes associated with the OUTDOOR intervention, specifically if there are any ways in which the processes could be improved.

8. To what extend was it acceptable to you for therapists to attend training in trial procedures?
    u. *Do you think this could be improved? If so, how?*

9. Could you briefly describe the measures that needed to be put in place at a service level to support delivery of the trial?
    - *Consider site set up (e.g. storage of paper based documentation), trial delivery, and close.*
    - *Do you think this could be improved?*

10. Did you experience any organisational challenges to enable ongoing support of the trial?
    a. *Consider staffing, time, costs, data entry, uploading of recordings, adverse event reporting, training new staff.*
    b. *If yes, could these be avoided in a future large scale trial?*

11. With regards to the intervention data collection logs that were provided, how useful were they as a prompt?
    - *Can you tell more?*
    - *Do you think there was anything missing from the intervention data collection logs?*

12. Do you have any additional comments regarding the OUTDOOR intervention?

Thank – you that brings us to the end of the interview.
